# Methylation of Methyl 4-Hydroxy-2-thioxo-1,2-dihydroquinoline-3-carboxylate: Synthetic, Crystallographic, and Molecular Docking Studies

**DOI:** 10.3390/molecules25184238

**Published:** 2020-09-16

**Authors:** Sergiy M. Kovalenko, Oleksandr G. Drushlyak, Svitlana V. Shishkina, Irina S. Konovalova, Illia O. Mariutsa, Natalya D. Bunyatyan, Dmitry V. Kravchenko, Vladimir V. Ivanov, Alexandre V. Ivachtchenko, Thierry Langer

**Affiliations:** 1 Department of Organic Chemistry, V. N. Karazin Kharkiv National University, 4 Svobody sq., 61077 Kharkiv, Ukraine; kovalenko.sergiy.m@gmail.com (S.M.K.); sveta@xray.isc.kharkov.com (S.V.S.); vivanov5001@gmail.com (V.V.I.); 2 ChemRar Research and Development Institute, Innovation Center Skolkovo territory, 7 Nobel st., 143026 Moscow, Russia; av@chemdiv.com; 3 Federal State Autonomous Educational Institution of Higher Education I.M. Sechenov First Moscow State Medical University of the Ministry of Healthcare of the Russian Federation, 8 Trubeckaya st., 119991 Moscow, Russia; ndbun@mail.ru; 4 Faculty of Pharmacy, The National University of Pharmacy, 53 Pushkinska st., 61002 Kharkiv, Ukraine; mariutsaillia@gmail.com; 5 SSI Institute for Single Crystals, National Academy of Sciences of Ukraine, 60 Nauky Ave, 61001 Kharkov, Ukraine; ikonovalova0210@gmail.com; 6 Federal State Budgetary Institution “Scientific Centre for Expert Evaluation of Medicinal Products” of the Ministry of Health of the Russian Federation, Petrovsky boulevard 8, bld. 2, 127051 Moscow, Russia; 7 Chemical Diversity Research Institute, 2A Rabochaya st., Khimki, 141400 Moscow, Russia; dk@chemrar.ru; 8 Department of Pharmaceutical Chemistry, University of Vienna, Althanstraße 14, A-1090 Vienna, Austria; thierry.langer@univie.ac.at

**Keywords:** quinoline, alkylation, X-ray analysis, hydrogen bond, molecular docking simulations, hepatitis B virus

## Abstract

Consecutive alkylation of 4-hydroxy-2-thioxo-1,2-dihydroquinoline-3-carboxylate by CH_3_I has been investigated to establish regioselectivity of the reaction for reliable design and synthesis of combinatorial libraries. In the first stage, the product of S-methylation-methyl 4-hydroxy-2-(methylthio)quinoline-3-carboxylate was obtained. The subsequent alkylation with CH_3_I led to the formation of both *O*- and *N*-methylation products mixture-methyl 4-methoxy-2-(methylthio)quinoline-3-carboxylate and methyl 1-methyl-2-(methylthio)-4-oxo-1,4-dihydroquinoline-3-carboxylate with a predominance of *O*-methylated product. The structure of synthesized compounds was confirmed by means of elemental analysis, ^1^H-NMR, ^13^C-NMR, LC/MS, and single-crystal X-ray diffraction. The quantum chemical calculations of geometry and electron structure of methyl 4-hydroxy-2-(methylthio)quinoline-3-carboxylate’s anion were carried out. According to molecular docking simulations, the studied compounds can be considered as potent inhibitors of Hepatitis B Virus replication. Experimental in vitro biological studies confirmed that studied compounds demonstrated high inhibition of HBV replication in 10 µM concentration.

## 1. Introduction

Derivatives of 4-hydroxyquinoline-3-carboxylic acid are known as highly effective quinolone antibiotics [1]. Their action is associated with inhibiting DNA gyrase and preventing duplication of bacterial DNA [2]. The presence of a sulfur atom in the 2 position of the quinoline moiety increases antibacterial activity [3,4,5,6,7,8,9]. Therefore, 4-hydroxy-2-thioxo-1,2-dihydroquinoline-3-carboxylates are interesting for the development of new effective bactericide compounds. Moreover, these compounds have recently attracted interest in other areas of their biological activity and are useful in the treatment of neurodegenerative diseases [10]. These compounds possess an anticancer activity due to inhibition of the c-Myc/Max/DNA complex formation [11,12].

**Figure 1:**
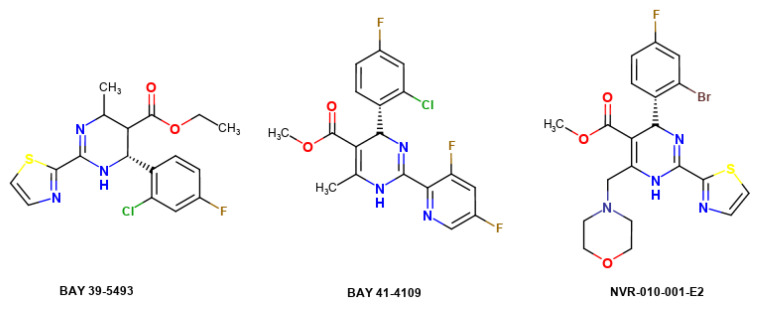
Heteroaryl dihydropyrimidines as the HBV inhibitors.

Nowadays, the main approach to creating anti-hepatitis B virus (HBV) agents is by developing a highly effective HBV replication depressor [13]. Heteroaryl dihydropyrimidines (HAP), such as BAY39-5493, BAY41-4109, NVR-010-001-E2 (Figure 1) are promising agents (HBV inhibitors) which have demonstrated interaction with the corresponding HBV core (HBcAg) proteins [14,15,16]. The PDB codes of HBV capsids are 5E0I, 5T2P, 5WRE and 5GMZ.

Those have demonstrated effective interactions with the corresponding HBV capsid and newly synthesized protein-nucleus and after the interaction of nucleus proteins, the protein could not be assembled properly.

Formerly we elaborated and described a new HBV infection model [17,18]. Derivatives of 4-hydroxyquinoline-3-carboxylic acid studied in the present paper fragmentally can be considered as the structural analogues of HAP systems. Therefore, it will be interesting to carry out molecular docking simulations for the quinoline-3-carboxylate derivatives and corresponding proteins, for example, the 5E0I complex.

**Scheme 1:**
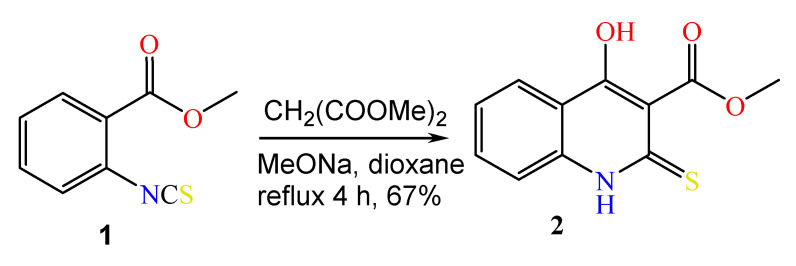
The reaction of methyl 2-isothiocyanatobenzoate **1** with dimethyl malonate.

Previously we developed a new simple method of one-pot synthesis of methyl 4-hydroxy-2-thioxo-1,2-dihydroquinoline-3-carboxylate **2** via condensation of methyl 2-isothiocyanatobenzoate **1** and methyl malonate [19] (Scheme 1).

The 4-hydroxy-2-thioxo-1,2-dihydroquinoline-3-carboxylate core has three reaction centers suitable for attack by an electrophilic reagent and could produce *S*-, *N*- and *O*-alkylated compounds. To establish the regioselectivity of the reaction with the purpose of reliable design and synthesis of combinatorial libraries of alkylated products, we investigated the alkylation of methyl 4-hydroxy-2-thioxo-1,2-dihydroquinoline-3-carboxylate **2** using an example of CH_3_I. Also, a preliminary evaluation of anti-hepatitis B virus activity of obtained quinoline derivatives was carried out by theoretical and experimental in vitro methods.

## 2. Results and Discussion

### 2.1. The Reaction of Alkylation of 4-Hydroxy-2-thioxo-1,2-dihydroquinoline-3-carboxylate **2** by CH_3_I

**Scheme 2:**
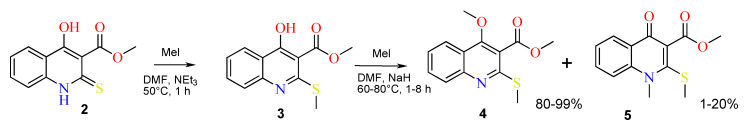
Consequent alkylation of 4-hydroxy-2-thioxo-1,2-dihydroquinoline-3-carboxylate **2** with CH_3_I.

At the first stage, alkylation of methyl 4-hydroxy-2-thioxo-1,2-dihydroquinoline-3-carboxylate **2** with CH_3_I was carried out under relatively mild conditions in DMF solution at 50 °C for 1 h with triethylamine as the base. The only product of *S*-methylation methyl 4-hydroxy-2-(methylthio)quinoline-3-carboxylate **3** was obtained with near quantitative yield as a raw product and more than 80% yield after crystallization. No traces of other products of alkylation were observed by TLC control of the reaction (Scheme 2).

The subsequent alkylation of 4-hydroxy-2-(methylthio)quinoline-3-carboxylate **3** with CH_3_I required a stronger base then triethylamine because the yield of alkylated product **4** did not exceed 3%. When the alkylation of 4-hydroxy-2-(methylthio)quinoline-3-carboxylate **2** by CH_3_I was carried out using a stronger base like NaH or K_2_CO_3_, the precipitate containing 2 products was formed. These pure substances were isolated by column chromatography with CHCl_3_ as eluent. The major product was methyl 4-methoxy-2-(methylthio)quinoline-3-carboxylate **4** with yield of 80–99%, and the minor was methyl 1-methyl-2-(methylthio)-4-oxo-1,4-dihydroquinoline-3-carboxylate **5** with a yield of 1–20% (Table 1). It should be noted that the practically pure *O*-methylated product **4** was obtained by prolonged heating (Table 1, cases 7–10), while a noticeable amount of *N*-methylated product **5** was formed with relatively short heating (Table 1, cases 5, 6)

**Scheme 3:**
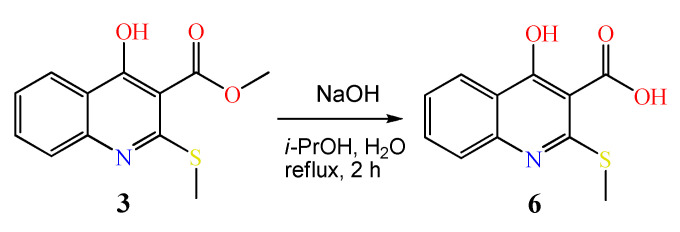
Synthesis of 4-hydroxy-2-(methylthio)quinoline-3-carboxylic acid **6**.

In addition, for investigation of their anti-HIV activity, 4-hydroxy-2-(methylthio)quinoline-3-carboxylic acid **6** was obtained from methyl 4-hydroxy-2-(methylthio)quinoline-3-carboxylate **3** by usual hydrolysis (Scheme 3).

The purity and structures of obtained products **3**–**6** were confirmed by LC/MS data, which presented a corresponding signal of [M + H]^+^ ion. Just S-alkylation was proved by the position of the singlet of SCH_3_ protons at 2.65 ppm in ^1^H-NMR spectrum of methyl 4-hydroxy-2-(methylthio)quinoline-3-carboxylate **3** and SCH_3_ carbon at 15.6 ppm in ^13^C-NMR spectrum. The broad peak of the OH proton was present at 11.60 ppm, but the signal of the thioamide proton near 13 ppm, that is characteristic for cyclic thioamides [19], was absent in ^1^H -NMR spectrum of the product **3**. In the ^1^H-NMR spectrum of methyl 4-methoxy-2-(methylthio)quinoline-3-carboxylate **4**, the new peak of the 4-OCH_3_ protons appeared at 4.03 ppm, while the peak of the OCH_3_ protons of ester group was shifted to 3.94 ppm. In ^1^H-NMR spectrum of methyl 1-methyl-2-(methylthio)-4-oxo-1,4-dihydroquinoline-3-carboxylate **5**, the new peak of the NCH_3_ protons appeared at 4.11 ppm, and the peak of the OCH_3_ protons of the ester group remained at 3.79 ppm. The second methylation did not significantly affect the position of the peak of SCH_3_ protons, which were slightly shifted to 2.62 and 2.54 ppm, respectively. The structure of 4-hydroxy-2-(methylthio)quinoline-3-carboxylic acid **6** was confirmed by the presence of broad singlets of the 4-OH protons at 11.10 ppm and the COOH protons at 16.44 ppm. The peak of the OCH_3_ protons of the ester group was absent, while the singlet of SCH_3_ protons remains at 2.52 ppm (The spectral data may be accessed in the Supplementary Materials).

The retention time R_t_ of methyl 4-hydroxy-2-(methylthio)quinoline-3-carboxylate **3** was 0.81 and its peak could overlap the peak of methyl 1-methyl-2-(methylthio)-4-oxo-1,4-dihydroquinoline-3-carboxylate **5** with R_t_ 0.75. But methyl 4-methoxy-2-(methylthio)quinoline-3-carboxylate **4** had R_t_ 1.08, and the peak was clearly different from the peaks of both starting material **3** and *N*-methylated product **5**. Hence, the molar content of methylated products **4** and **5** in the reaction mixture could be estimated by the LCMS analysis of the crude product by the ratio of the integral intensity of peaks of products **4** and **5** providing low content of starting compound **3**, but the difference of extinction at 216 nm and 242 nm decreased the accuracy of estimation. In the case of noticeable content of *N*-methylated compound **5**, the molar content of reaction products could be evaluated by analysis of ^1^H-NMR spectra of crude products by the ratio of the integral intensity of the sharp singlets of the NCH_3_ protons at 4.11 ppm and OCH_3_ protons at 4.03 ppm to sum of integral intensities of the peaks of SCH_3_ protons appear at 2.55–2.65 ppm (Table 1).

The obtained results (Table 1) convincingly show that the methylation of methyl 4-hydroxy-2-(methylthio)quinoline-3-carboxylate **3** proceeds exclusively with the anion, which was generated by NaH or K_2_CO_3_ via the SN_2_ mechanism. If the basicity was insufficient for anion generation (triethylamine), the reaction did not pass to a noticeable degree. As a typical SN_2_ reaction, the reaction accelerates with increasing polarity of the solvent.

**Scheme 4:**
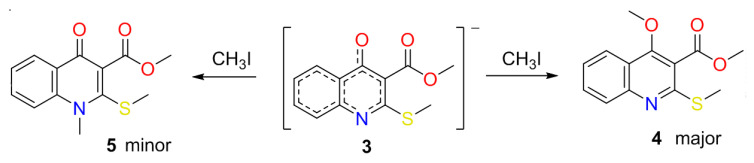
Alkylation of 4-hydroxy-2-thioxo-1,2-dihydroquinoline-3-carboxylate anion **3** by CH_3_I.

The observed direction of methylation of 4-hydroxy-2-(methylthio)quinoline-3-carboxylate **3** is in good agreement with the evaluation of electron distribution. The experimentally proved direction of the methylation process (Scheme 4) has been theoretically interpreted by using DFT B3LYP/cc-pVDZ calculation of geometry and electron structure of the corresponding anion. All the quantum chemical calculations were carried out using Gaussian 09 [20].

The system with the lowest energy corresponds to the geometrical configuration of SCH_3_ group rotated in a direction to the nitrogen atom. This picture is in a good agreement with the crystal structure of compound **3** (see the next section). The closest higher energy conformation, in which the SCH_3_ group is rotated in the direction of the OCH_3_ group, differs from the lowest energy conformation by 7 kcal/mol.

**Figure 2:**
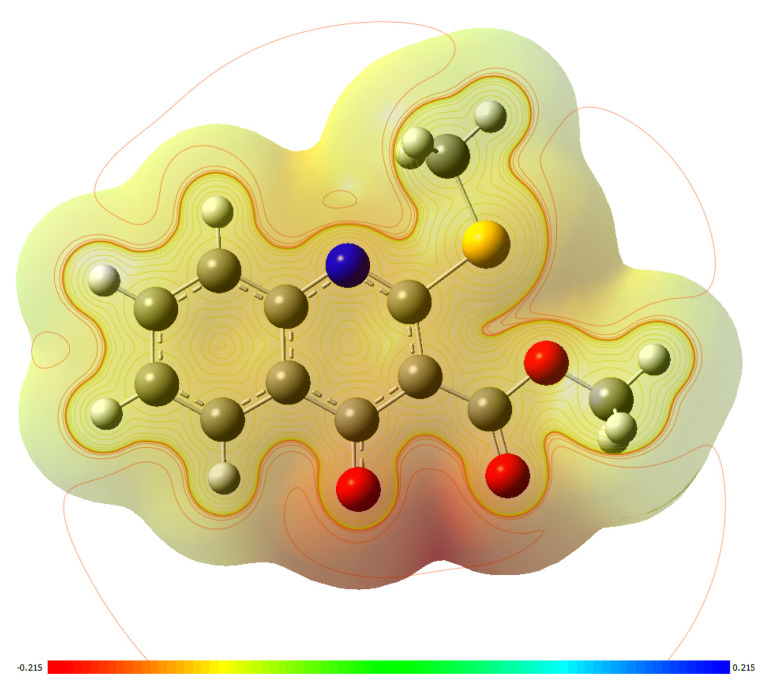
Visualization of the total electrostatic potential map of anion **3** (red regions corresponds to negative charge).

The obtained electronic distribution demonstrated equivalent atomic net charges for both nitrogen and oxygen atoms of 3-(methoxycarbonyl)-2-(methylthio)-1,2-dihydroquinolin-4-olate anion fragment. According to Bader, the analysis we obtained had the same values of atomic charges as −1.19 e. However, the calculated 3D electrostatic potential map (Figure 2) demonstrated a significant and rather large region of electron density near the oxygen atom for the state under consideration.

**Figure 3:**
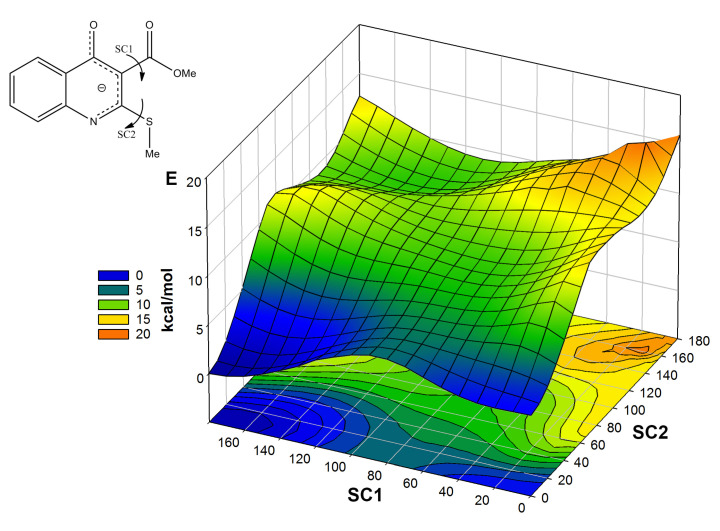
The Csp2–Csp2–Csp2=O and N–Csp2–S–Csp3 torsion angles used for SC1 and SC2 scanning during the study of potential energy surface (PES) in conformational analysis of 3-(methoxycarbonyl)-2-(methylthio)-1,2-dihydroquinolin-4-olate anion; PES as a function of energy and two scanned torsion angles SC1 and SC2.

**Figure 4:**
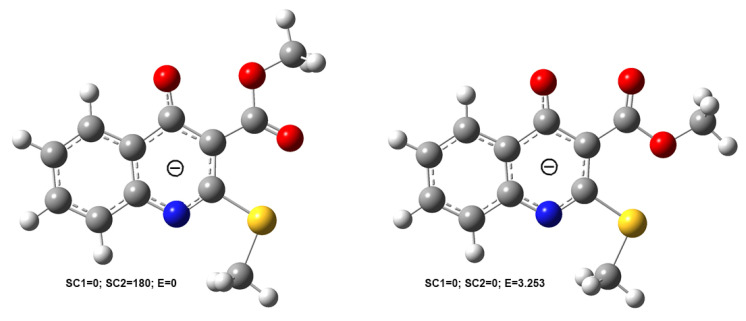
The most stable conformers of 3-(methoxycarbonyl)-2-(methylthio)-1,2-dihydroquinolin- 4-olate anion according to the conformational study.

Additionally, the preferable methylation of the oxygen atom as compared to the nitrogen atom may be caused by steric factors. To determine all stable conformations of 3-(methoxycarbonyl)-2-(methylthio)-1,2-dihydroquinolin-4-olate anion, we performed the study of full potential energy surface (PES) constructed as a function of energy and two torsion angles (SC1 and SC2 at Figure 3). The results of the conformational study showed that two of the most stable conformers differ by the rotation of the ester group only (Figure 4). The methyl group at the Sulphur atom was found in sp-conformation to the N–C endocyclic bond shielding the nitrogen atom in the SN_2_ reaction.

As a result, despite the approximately equal negative charge of the nitrogen and the oxygen atom of the anion (**3**) (Figure 2), the distinct steric hindrance near the nitrogen atom led to the mostly methylation of the oxygen atom.

### 2.2. X-ray Analysis of the Reaction Products

**Figure 5:**
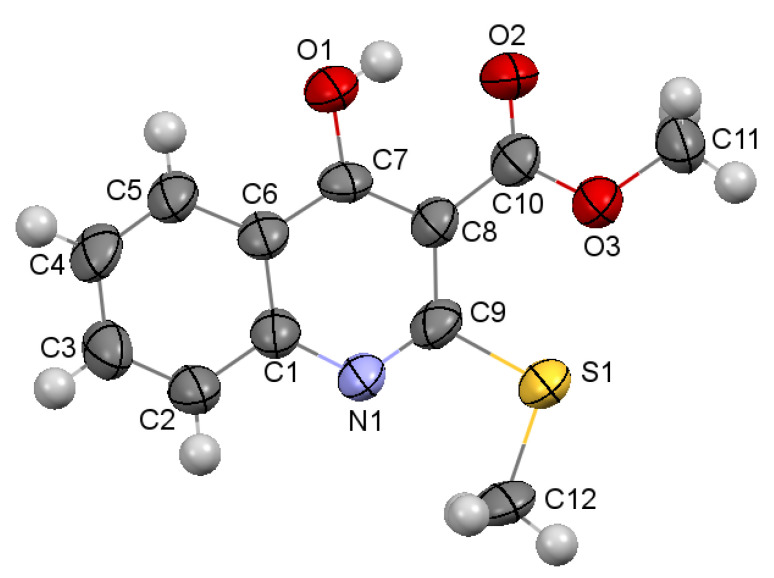
Molecular structure of compound **3** according to X-ray diffraction data. Thermal displacement ellipsoids are shown at the 50% probability level.

**Figure 6:**
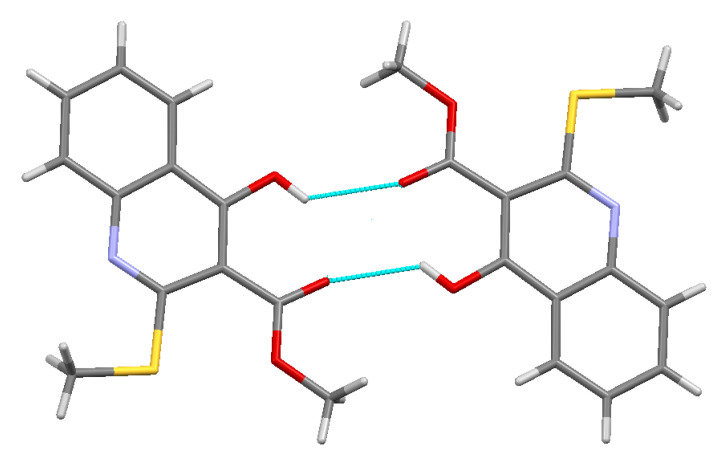
Centrosymmetric dimer of molecules **3** in the crystal phase. Hydrogen bonds are shown by dashed blue lines.

**Figure 7:**
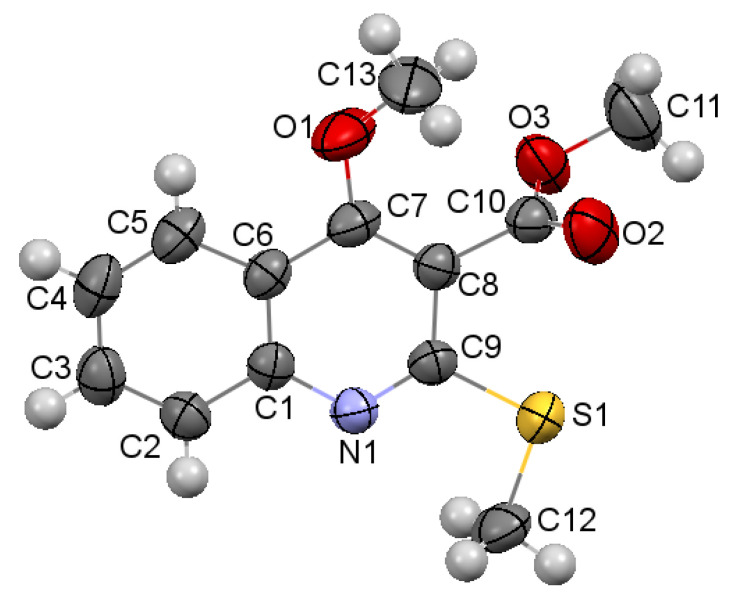
Molecular structure of compound **4** according to X-ray diffraction data. Thermal displacement ellipsoids are shown at the 50% probability level.

**Figure 8:**
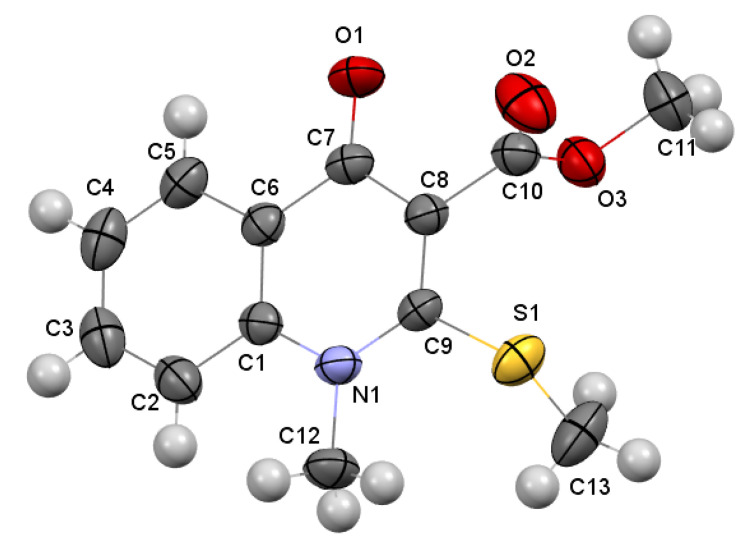
Molecular structure of compound **5** according to X-ray diffraction data. Thermal displacement ellipsoids are shown at the 50% probability level.

Finally, the structures of synthesized compounds **3**–**5** were confirmed by X-ray analysis (Figure 5,Figure 6,Figure 7,Figure 8).

All non-hydrogen atoms of molecule **3** lie within the plane with an accuracy of 0.04 Å despite the strong steric repulsion between the O3 atom of the ester substituent and the sulfur atom (the distance was 2.69 Å as compared to the van der Waals radii sum [21] 3.11 Å). The planar conformation was stabilized by the O1-H···O strong enough intramolecular hydrogen bond (H···O 1.85 Å, O-H···O 141°). The methyl group of the ester substituent was localized in ap-conformation to the C8-C10 bond (the C8-C10-O3-C11 torsion angle is 178.5(5)°) (Figure 5).

In the crystal phase, molecules **3** formed centrosymmetric dimers due to the O1-H···O2′ intermolecular hydrogen bonds (−x, 1 − y, 1 − z symmetry operation, H···O 2.40 Å, O-H···O 124°) (Figure 6).

The replacement of the hydroxyl group hydrogen atom by the methyl group (Figure 7) led to the appearance of additional steric repulsion between vicinal substituents (the shortened intramolecular contacts were C13···C10 2.95 Å, H13c···C10 2.64 Å, H13a···C10 2.78 Å as compared to van der Waals radii sum [21] C···C 3.42 Å and H···C 2.87 Å). As a result, the ester group and the methyl group of the methoxy substituent were turned relative to the C7-C8 endocyclic double bond (the C7-C8-C10-O2 torsion angle is 104.4(3)°, the C8-C7-O1-C13 torsion angle was −30.2(4)°). The methyl group of the ester substituent was located in ap-conformation to the C8-C10 bond similar to molecule **2** (the C8-C10-O3-C11 torsion angle is −178.7(2)°). The methyl substituent at the S1 atom was coplanar to the bicyclic plane (the N1-C9-S1-C12 torsion angle is 5.2(2)°).

The introduction of the methyl substituent to the cyclic nitrogen atom (Figure 8) resulted in the redistribution of electron density within the heterocycle and created the strong repulsion with the sulfur atom as well as with atoms of the aromatic cycle (the short intramolecular contacts H12c···S1 2.51 Å (3.00 Å), H12b···H2 2.14 Å (2.32 Å), H12b···C2 2.64 Å (2.87 Å), H2···C12 2.49 Å (2.87 Å)). Such a repulsion caused the orthogonal orientation of the methyl substituent at the S1 atom relatively to the N1-C9 endocyclic bond (the N1-C9-S1-C13 torsion angle is 78.8(2)°). The ester group was located practically in an orthogonal position relative to the C7-C8 endocyclic double bond (the C7-C8-C10-O2 torsion angle is 85.9(2)°), and the methyl group was located in ap-conformation to the C8-C10 bond (the C8-C10-O3-C11 torsion angle is 175.6(2)°). The carbonyl C7–O1 bond (1.245(2) Å) is some elongated as compared with the average value for C=O bond (1.210 Å) [22].

**Figure 9:**
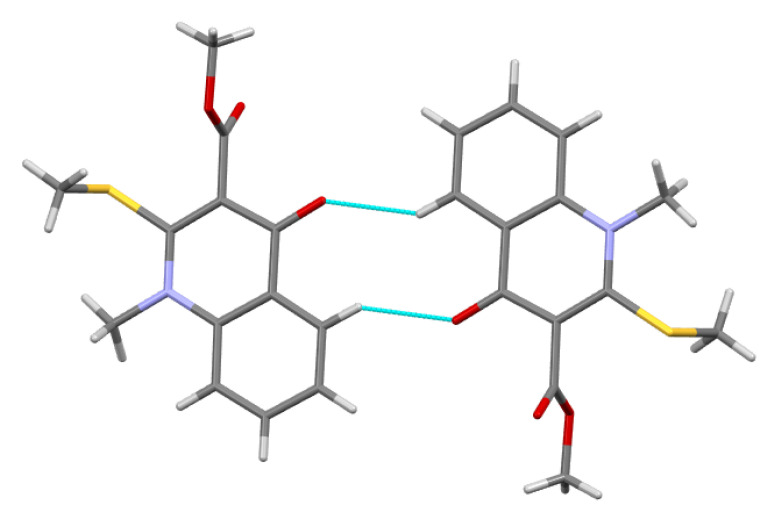
Centrosymmetric dimer of molecules **5** in the crystal phase. Hydrogen bonds are shown by dashed blue lines.

**Figure 10:**
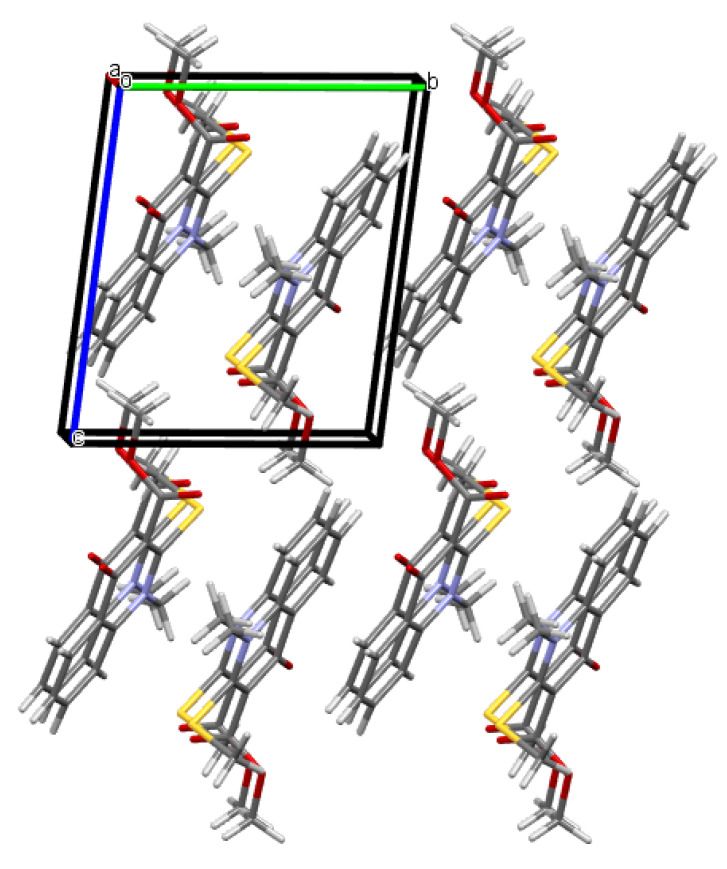
Packing of molecules **5** in the crystal. The projection along the [1 0 0] crystallographic direction is presented.

In the crystal phase, molecules **5** form centrosymmetric dimers due to the weak C5-H···O1′ intermolecular hydrogen bonds (−x, −y, 1 − z symmetry operation, H···O 2.57 Å, C-H···O 141°) (Figure 9). These dimers were packed as a double column due to the stacking interactions (−2 + x, −1 + y, z symmetry operation, C···C 3.39 Å) (Figure 10).

### 2.3. Molecular Docking Simulations

The presented set of molecules were tested as HBV (hepatitis B virus) capsid inhibitors. The molecular structure of these systems can be obtained by using different molecular graphical program, for instance Jmol [23]. Experimental X-ray crystallographic data for corresponding “HAP-protein” complexes were taken from [24]. There are several equivalent proteins available (pdb codes of complexes are: 5T2P, 5WRE, 5GMZ, 5E0I). In our simulation, we used 5E0I complex.

The extraction pharmacophorus and the in silico docking simulation were performed by using Ligandscout 4.4. program complex [25]. From six chains of protein lines (designated as A, B, C, D, E, and F), the D chain was selected for simulation. According to our calculations for the D-chain redocking procedure of reference (core) molecule (HAP), characterized minimal value root mean squared (RMSD) value, namely for the D-chain, the RMSD value was small as <1 Å.

**Figure 11:**
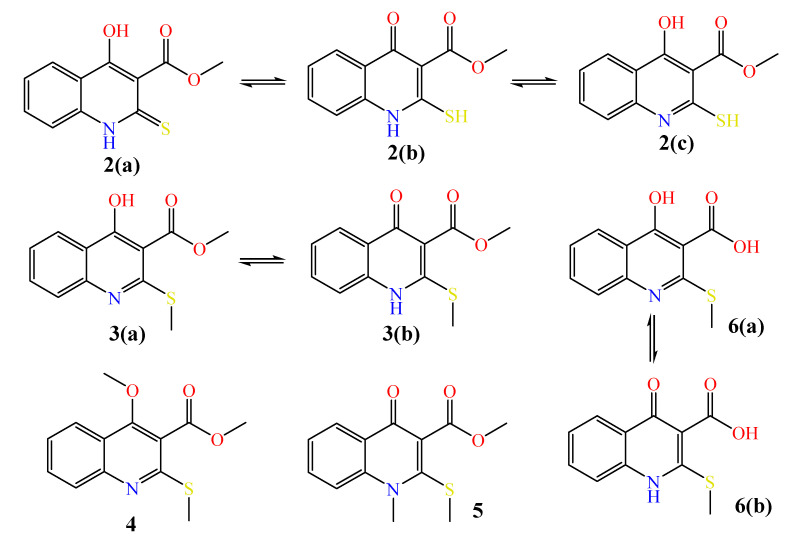
The molecular systems (**2**–**6**) under consideration for the docking procedure.

**Figure 12:**
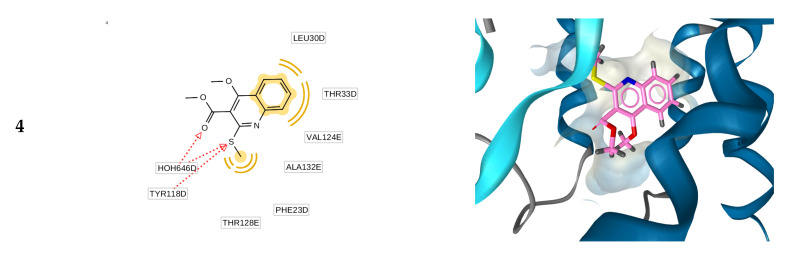

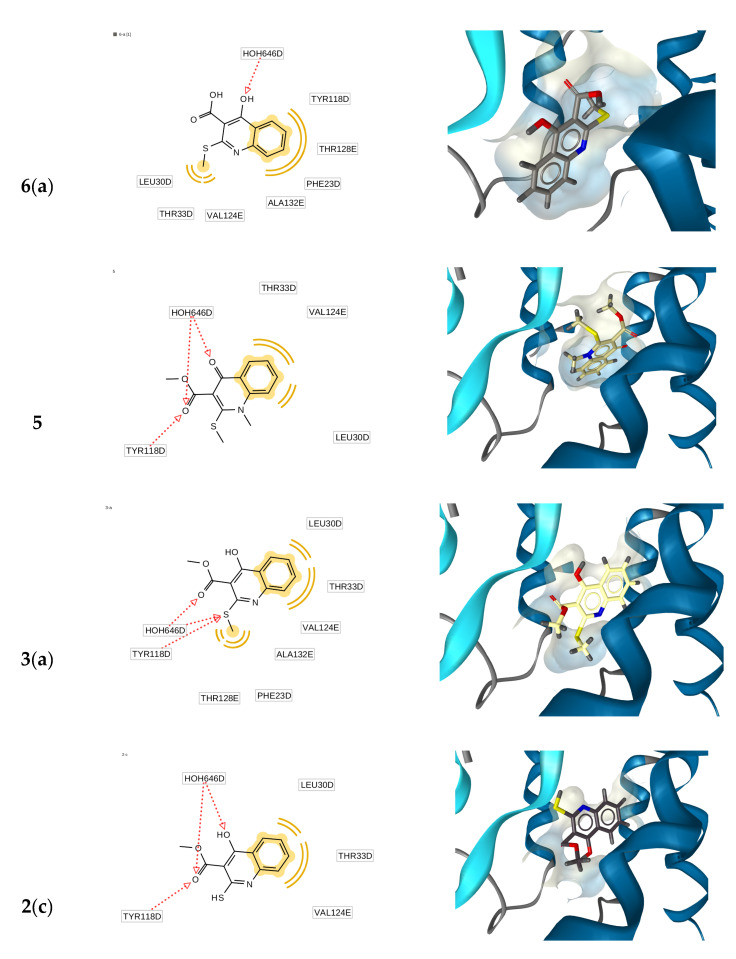
Complexes with highest affinity score presented according to Table 2.

The screening procedure for this set of molecules (Figure 11) gives the possibility to select systems with the highest pharmacofore fit score. Figure 11 contains all necessary tautomer forms of systems under consideration. For those molecules, docking procedures were performed. The graphical results with the highest binding affinity score (BAS) and binding energy (BE, kcal/mol) of the docking procedure are presented in Figure 12. Here the two-dimensional representations (2D) correspond to the “pharmacoforic picture” of interaction between ligand and protein, where the dotted red lines designate hydrogen bonds while yellow ones are hydrophobic interaction.

Corresponding molecular parameters are presented in Table 2. Among binding affinity parameters, there are also lipophilicity (cLogP) and topological molecular polar surface area (TPSA), calculated according to [26]. These parameters are important characteristics of transport through membranes properties of the drug. As can been seen, all the considered systems demonstrated a high level of affinity to protein comparatively to the reference system. Hence the discussed systems can be treated as a potent inhibitor of HBV replications.

### 2.4. Anti-Hepatitis B Virus (HBV) Activity

Biological activity of compounds **3**, **4**, and **6** were studied using an experimental in vitro hepatitis B virus infection model based on human hepatoma line HepG2 stably transfected with the NTCP gene [27]. This model, which maintains a full virus replication cycle, was developed in our laboratories for the identification of viral entry inhibitors, promising candidates to prevent the development of resistant HBV forms [17]. Cytotoxicity of the tested compounds, as cell survival percentage, was also measured. The molecules **3**, **4**, and **6** demonstrated inhibition of HBV replication (in 10 µM concentration) depicted in Table 3.

Taking into account the recently reported test results for viral entry inhibition of FDA approved drugs zafirlukast (IC_50_ 6.5 µM), TRIAC (IC_50_ 6.9 µM), and sulfasalazine (IC_50_ 9.6 µM) [28] HBV inhibition demonstrated in Table 3 (which is in accordance with docking results) in combination with low cytotoxicity of the tested items let us consider the compounds **3**, **4** and **6** as promising candidates for further investigations in this area.

## 3. Materials and Methods

### 3.1. General Information

All NMR spectra were recorded on a Varian MR-400 spectrometer (Varian, Inc., Walnut Creek, CA, USA) with standard pulse sequences operating at 400 MHz for 1H-NMR and 100 MHz for 13C-NMR. For all NMR spectra, DMSO-d6 was used as a solvent. Chemical shift values are referenced to residual protons (δ 2.49 ppm) and carbons (δ 39.6 ppm) of the solvent as an internal standard. Elemental analysis was performed on a EuroEA-3000 CHNS-O analyzer (Euro Vector, Milan, Italy). Melting points were measured with a Buchi B-520 melting point apparatus (Buchi AG, Flawil, Switzerland). LC/MS spectra were recorded with ELSD Alltech 3300 liquid chromatograph (Buchi AG, Flawil, Switzerland) equipped with a UV detector (λ_max_ 254 nm), API-150EX mass-spectrometer and using a Zorbax SB-C18 column, Phenomenex (100 × 4 mm) Rapid Resolution HT Cartridge 4.6 × 30 mm, 1.8-Micron. Elution started with 0.1 M solution of HCOOH in water and ended with 0.1 M solution of HCOOH in acetonitrile used a linear gradient at a flow rate of 0.15 mL/min and an analysis cycle time of 25 min. UV/Vis spectra of solutions in CH_3_CN were recorded on a Specord 200 spectrometer (Analytik Jena AG, Jena, Germany). IR spectra in KBr pellets were recorded on a Bruker Vertex 70 FTIR spectrometer (Bruker Optik GmbH, Ettlingen, Germany).

### 3.2. Synthesis

Starting methyl 4-hydroxy-2-thioxo-1,2-dihydroquinoline-3-carboxylate **2** was obtained according to method [19]. CH_3_I, NaH, K_2_CO_3_ and solvents are commercially available, were reagent grade and were used without further purification. Silica gel (40–60 μm) from Merck was used for column chromatography.

#### 3.2.1. Synthesis of Methyl 4-Hydroxy-2-(methylthio)quinoline-3-carboxylate **3**

To the stirred solution of methyl 4-hydroxy-2-thioxo-1,2-dihydroquinoline-3-carboxylate **2** (2.35 g, 10 mmol) and triethylamine (1.5 mL, 12 mmol) in anhydrous DMF (20 mL) MeI (0.7 mL, 11 mmol) was added. The reaction mixture was heated at 50 °C for 1 h. After cooling, the reaction mixture was diluted with water (50 mL). The precipitate that formed was filtered, washed with MeOH (10 mL) and recrystallized from the mixture of DMF (10 mL) and MeOH (30 mL). Yield 2.02 g (81%), white solids, m.p. 181–182 °C. ^1^H-NMR spectrum δ, ppm (*J*, Hz): 11.60 (br. s, 1H, OH), 8.05 (d, *J* = 7.6, 1H, H Ar), 7.72–7.64 (m, 2H, H Ar), 7.36 (td, *J* = 7.6, *J* = 1.1, 1H, H Ar), 3.77 (s, 3H, OCH_3_), 2.65 (s, 3H, SCH_3_). ^13^C-NMR spectrum, δ, ppm: 172.0 (2-CS), 166.5, 149.2, 140.6, 132.4 (2 C), 124.7, 124.2 (2 C), 118.4, 52.0 (COOCH_3_), 15.6 (SCH_3_). LC/MS *m*/*z* (%): 250.2 [M + H]^+^ (90.0), 218.2 (100). Anal. calcd. for C_12_H_11_NO_3_S, %: C 57.82; H 4.45; N 5.62; S 12.86. Found, %: C 57.72; H 4.43; N 5.63; S 12.94.

#### 3.2.2. Reaction of Methyl 4-Hydroxy-2-(methylthio)quinoline-3-carboxylate **3** with CH_3_I with the Presence of Sodium Hydride

To the stirred solution of methyl 4-hydroxy-2-(methylthio)quinoline-3-carboxylate **3** (0.5 g, 2 mmol) in anhydrous DMF (10 mL) 60% dispersion of NaH in mineral oil (0.088 g, 2.2 mmol) was added. Then CH_3_I (0.07 mL, 2.2 mmol) was added, and the reaction mixture was heated at 60–80 °C for 1–8 h (Table 1). The reaction mixture was diluted with water (50 mL) and extracted with CHCl_3_ (10 mL) twice. The extracts were washed with water (10 mL) thrice and dried over anhydrous Na_2_SO_4_. Then the mixture was purified by column chromatography (CHCl_3_) to give the products **4** and **5**. The analytical data for representative compounds are shown below. ^1^H-NMR and ^13^C-NMR spectra of compounds **4** and **5** are presented in Supplementary Materials.

*Methyl 4-methoxy-2-(methylthio)quinoline-3-carboxylate* (**4**), yield of 0.42 g (80%), white solids, m.p. 100–101 °C. ^1^H-NMR spectrum δ, ppm (*J*, Hz): 8.09 (dd, *J* = 7.6, *J* = 1.1, 1H, H Ar), 7.89 (dd, *J* = 7.6, *J* = 1.1, 1H, H Ar), 7.80 (td, *J* = 7.6, *J* = 1.1, 1H, H Ar), 7.56 (td, *J* = 7.6, *J* = 1.1, 1H, H Ar), 4.03 (s, 3H, 4-OCH_3_), 3.94 (s, 3H, COOCH_3_), 2.62 (s, 3H, SCH_3_). ^13^C-NMR spectrum, δ, ppm: 166.0 (2-CS), 159.8, 157.2, 148.3, 131.5, 127.5, 125.8, 122.7, 119.8, 114.6, 61.5 (4-OCH_3_), 52.9 (COOCH_3_), 13.0 (SCH_3_). LC/MS *m*/*z* (%): 264.2 [M + H]^+^ (100.0), 232.2 (50). Anal. calcd. for C_13_H_13_NO_3_S %: C 59.30; H 4.98; N 5.32; S 12.18. Found, %: C 59.22; H 5.01; N 5.26; S 12.11.

*Methyl 1-methyl-2-(methylthio)-4-oxo-1,4-dihydroquinoline-3-carboxylate* (**5**), yield of 0.104 g (20%), white solids, m.p. 169–171 °C. ^1^H-NMR spectrum δ, ppm (*J*, Hz): 8.16 (dd, *J* = 7.6, 1H, *J* = 1.1, H Ar), 7.88–7.81 (m, 2H, H Ar), 7.48 (td, *J* = 7.6, *J* = 1.1, 1H, H Ar), 4.11 (s, 3H, 1-NCH_3_), 3.79 (s, 3H, COOCH_3_), 2.54 (s, 3H, SCH_3_). ^13^C-NMR spectrum, δ, ppm: 172.4 (2-CS), 166.1, 148.1, 141.9, 133.2, 125.9, 125.3, 124.3, 124.2, 118.2, 52.1 (COOCH_3_), 36.9 (1-NCH_3_), 19.0 (SCH_3_). LC/MS *m*/*z* (%): 264.0 [M + H]^+^ (90.0), 232.0 (100.0). Anal. calcd. for C_13_H_13_NO_3_S %: C 59.30; H 4.98; N 5.32; S 12.18. Found, %: C 59.46; H 5.00; N 5.29; S 12.21.

#### 3.2.3. Reaction of Methyl 4-Hydroxy-2-(methylthio)quinoline-3-carboxylate **3** with CH_3_I with the Presence of K_2_CO_3_

To the stirred solution of methyl 4-hydroxy-2-(methylthio)quinoline-3-carboxylate **3** (0.5 g, 2 mmol) in corresponding solvent (acetone, DMF, DMSO) (Table 1) (10 mL) powder of K_2_CO_3_ (0.83 g, 6 mmol) was added. Then CH_3_I (0.07 mL, 2.2 mmol) was added, and the reaction mixture was heated at 60–80 °C for 1–8 h (Table 1). The reaction mixture was diluted with water (50 mL) and extracted with CHCl_3_ (10 mL) twice. The extracts were washed with water (10 mL) thrice and dried over anhydrous Na_2_SO_4_. Then the mixture was purified by column chromatography (CHCl_3_) to yield the products **4** and **5**.

#### 3.2.4. Synthesis of 4-Hydroxy-2-(methylthio)quinoline-3-carboxylic acid **6**

The stirred solution of methyl 4-hydroxy-2-(methylthio)quinoline-3-carboxylate **3** (0.25 g, 1 mmol) in a mixture of *i*-PrOH (5 mL) and 3 mL of 1 M water solution of NaOH (0.12 g, 3 mmol) was refluxed for 2 h. After cooling, the reaction mixture was acidified to pH 6 by a solution of AcOH (0.3 mL, 5 mmol) in 10 mL of water. The precipitate that formed was filtered, washed twice with water (5 mL) and recrystallized from a mixture of DMF (5 mL) and water (10 mL). Yield 0.16 g (68%), white solids, m.p. 190 °C (decomposition). ^1^H-NMR spectrum δ, ppm (*J*, Hz): 16.44 (br. s, 1H, COOH), 11.10 (br. s, 1H, 4-OH), 8.22 (d, *J* = 7.6, 1H, H Ar), 8.07 (d, *J* = 7.6, 1H, H Ar), 7.85 (t, *J* = 7.6, 1H, H Ar), 7.53 (t, *J* = 7.6, 1H, H Ar), 2.52 (s, 3H, SCH_3_). ^13^C-NMR spectrum, δ, ppm: 176.7 (2-CS), 166.8, 163.0, 139.2, 133.6, 125.7, 124.9, 121.8, 119.0, 105.2, 14.4 (SCH_3_). LC/MS *m*/*z* (%): 236.0 [M + H]^+^ (50.0), 218.0 (100). Anal. calcd. for C_11_H_9_NO_3_S, %: C 56.16; H 3.86; N 5.95; S 13.63. Found, %: C 55.97; H 3.88; N 5.93; S 13.59.

### 3.3. X-ray Diffraction Study

#### 3.3.1. Methyl 4-Hydroxy-2-(methylthio)quinoline-3-carboxylate **3**

Single crystals for X-ray diffraction study were grown from MeOH. The colorless crystals of **3** (C_12_H_11_NO_3_S) are monoclinic. At 293 °K *a* = 4.0161(9) Å, *b* = 17.850(4) Å, *c* = 15.891(6) Å, β = 96.13(3)°, *V* = 1132.6(5(2) Å^3^, M_r_ = 249.28, Z = 4, space group P2_1_/c, d_calc_ = 1.462 g/cm^3^, μ(MoK_α_) = 0.280 mm^−1^, F(000) = 520. Intensities of 12582 reflections (7965 independent, R_int_ = 0.166) were measured on the ”Xcalibur-3” diffractometer (graphite monochromated MoK_α_ radiation, CCD detector, ω-scaning, 2θ_max_ = 50°). The structure was solved by direct method using SHELXTL package [29]. Positions of the hydrogen atoms were located from electron density difference maps and refined by “riding” model with U_iso_ = *n*U_eq_ (*n* = 1.5 for methyl group and *n* = 1.2 for other hydrogen atoms) of the carrier atom. Full-matrix least-squares refinement against F^2^ in anisotropic approximation for non-hydrogen atoms using 1960 reflections was converged to wR_2_ = 0.177 (R_1_ = 0.084 for 819 reflections with F > 4σ(F), S = 0.874). The final atomic coordinates, and crystallographic data for molecule **3** have been deposited to with the Cambridge Crystallographic Data Centre, 12 Union Road, CB2 1EZ, UK (fax: +44-1223-336033; e-mail: deposit@ccdc.cam.ac.uk) and are available on request quoting the deposition numbers CCDC 1982129).

#### 3.3.2. Methyl 4-Methoxy-2-(methylthio)quinoline-3-carboxylate **4**

Single crystals for X-ray diffraction study were grown from MeOH. The colorless crystals of **4** (C_13_H_13_NO_3_S) are monoclinic. At 293 °K *a* = 9.5401(7) Å, *b* = 11.8332(8) Å, *c* = 11.858(1) Å, β = 109.436(8)°, *V* = 1262.3(2) Å^3^, M_r_ = 263.3, Z = 4, space group P2_1_/c, d_calc_= 1.385 g/cm^3^, μ(MoK_α_) = 0.256 mm^−1^, F(000) = 552. Intensities of 13980 reflections (8643 independent, R_int_ = 0.072) were measured on the ”Xcalibur-3” diffractometer (graphite monochromated MoK_α_ radiation, CCD detector, ω-scaning, 2θ_max_ = 50°). The structure was solved by direct method using SHELXTL package [29]. Positions of the hydrogen atoms were located from electron density difference maps and refined by “riding” model with U_iso_ = *n*U_eq_ (*n* = 1.5 for methyl group and *n* = 1.2 for other hydrogen atoms) of the carrier atom. Full-matrix least-squares refinement against F^2^ in anisotropic approximation for non-hydrogen atoms using 2160 reflections was converged to wR_2_ = 0.123 (R_1_ = 0.049 for 1541 reflections with F > 4σ(F), S = 0.998). The final atomic coordinates, and crystallographic data for molecule **4** have been deposited to with the Cambridge Crystallographic Data Centre, 12 Union Road, CB2 1EZ, UK (fax.: +44-1223-336033; e-mail: deposit@ccdc.cam.ac.uk) and are available on request quoting the deposition numbers CCDC 1982132).

#### 3.3.3. Methyl 1-Methyl-2-(methylthio)-4-oxo-1,4-dihydroquinoline-3-carboxylate **5**

Single crystals for X-ray diffraction study were grown from MeOH. The colorless crystals of **5** (C_13_H_13_NO_3_S) are triclinic. At 293 °K *a* = 7.8907(9) Å, *b* = 8.2795(9) Å, *c* = 9.8449(12) Å, α = 96.170(9)°, β = 102.146(10)°, γ = 98.598(9)°, *V* = 615.26(13) Å^3^, M_r_ = 263.3, Z = 2, space group P$\bar{1}$, d_calc_= 1.421 g/cm^3^, μ(MoK_α_) = 0.262 mm^−1^, F(000) = 276. Intensities of 6402 reflections (4189 independent, R_int_ = 0.040) were measured on the “Xcalibur-3” diffractometer (graphite monochromated MoK_α_ radiation, CCD detector, ω-scaning, 2θ_max_ = 50°). The structure was solved by direct method using SHELXTL package [29]. Positions of the hydrogen atoms were located from electron density difference maps and refined by “riding” model with U_iso_ = *n*U_eq_ (*n* = 1.5 for methyl group and *n* = 1.2 for other hydrogen atoms) of the carrier atom. Full-matrix least-squares refinement against F^2^ in anisotropic approximation for non-hydrogen atoms using 2164 reflections was converged to wR_2_ = 0.127 (R_1_ = 0.045 for 1820 reflections with F > 4σ(F), S = 1.037). The final atomic coordinates, and crystallographic data for molecule **5** have been deposited to with the Cambridge Crystallographic Data Centre, 12 Union Road, CB2 1EZ, UK (fax: +44-1223-336033; e-mail: deposit@ccdc.cam.ac.uk) and are available on request quoting the deposition numbers CCDC 1982133).

### 3.4. Anti-Hepatitis B Virus (HBV) Activity

The final stage of our investigation was the experimental study of the biological activity of synthesized molecules (**3**, **4**, and **6**). The human hepatoma cell line HepAD38 was chosen as the source of infectious viral particles to infect the HepG2 / NTCP cell line, carrying the stable integrated HBV virus gene under the control of a tetracycline-regulated promoter, and secreting viral particles into the culture medium in the absence of tetracycline. HBV preparation was obtained using the HepAD38 line according to the following protocol: HepAD38 cells were passaged in a DMEM medium containing 10% fetal calf serum, penicillin/streptomycin, and essential amino acids. The culture medium was taken once every 2 days, clarified by centrifugation (200× *g*, 15 min) and stored at 4 °C for no longer than 7 days. Next, dry PEG 8000 was added to the culture media to a final concentration of 7.5% and incubated at 4 °C on a rotary platform overnight. The viral precipitate was separated by centrifugation (2000× *g*, 30 min), and the precipitate was suspended in 1/100 of the initial volume in OPTI-MEM medium. Thus obtained viral preparation was aliquoted and stored at −80 °C.

Infection was carried out as follows: The HepG2-NTCP cell suspension was distributed to 96-well plates at 2000 cells per well. After the cells were attached (on the same or the next day), the initial solution was removed by aspiration, and 50 μL of a solution of test compounds dissolved in OPTI-MEM medium (with a final DMSO concentration of 2%) was added to each well or OPTI-MEM with 2% DMSO (in the wells of the positive and negative controls of the infection) and 50 µL of the HBV preparation diluted in OPTI-MEM with 2% DMSO (except negative infection control). After incubation for 24 h in a humidified atmosphere containing 5% CO_2_, the HBV medium was removed by aspiration, and 200 μL of DMEM culture medium containing the corresponding test compounds in 10 mkM concentration was added to the cultures. The cells were additionally incubated for 6 days at 37 °C in a humidified atmosphere containing 5% carbon dioxide. Next, cell supernatants (50 μL) were analyzed for viral antigen content using a commercial HBeAg ELISA 4.0 kit (Creative Diagnostics, catalog number DEIA003) according to the kit manufacturer’s protocol and the optical density of each analyzed well was measured at a wavelength of 450 nm using a plate densitometer.

## 4. Conclusions

Alkylation of 4-hydroxy-2-thioxo-1,2-dihydroquinoline-3-carboxylate by CH_3_I primarily leads to the product of S-methylation methyl 4-hydroxy-2-(methylthio)quinoline-3-carboxylate. The subsequent alkylation with CH_3_I gives the mixture of products both *O*- and *N*-methylation – methyl 4-methoxy-2-(methylthio)quinoline-3-carboxylate and methyl 1-methyl-2-(methylthio)-4-oxo-1,4-dihydroquinoline-3-carboxylate with predominance of O-methylated product. The established regioselectivity of the reaction can be a good basis for further design and targeted synthesis of focused libraries of alkylated quinolines. According to Molecular Docking Simulations and experimental in vitro biological studies, the discussed compounds can be considered as potent inhibitors of HBV replication.

## Figures and Tables

**Table 1:** The reaction conditions for methylation of compound **3**.

Case	Solvent	Base	Temperature (°C)	Reaction Time (h)	Yield of 4 (%), Estimated by ^1^H-NMR	Yield of 5 (%), Estimated by ^1^H-NMR
1	DMF	NEt_3_	80	8	3 ^1^	0
2	DMF	DBU	80	8	7 ^1^	0
3	DMSO	DBU	80	8	25 ^1^	0
4	acetone	K_2_CO_3_	60	8	55 ^1^	5 ^1^
5	DMF	NaH	60	2	87	13
6	DMF	NaH	80	1	80	20
7	DMF	K_2_CO_3_	80	4	99	1
8	DMSO	K_2_CO_3_	80	2	99	0.8
9	DMF	NaH	80	8	99	0.4
10	DMF	K_2_CO_3_	80	8	99	0.6

^1^ The rest was starting compound **3**.

**Table 2:** Molecular Docking Parameters for the systems under consideration.

Mol	BAS	Final Intermolecular Energy (kcal/mol)	Est. Binding Energy (kcal/mol)	cLogP	TPSA [26]
reference	−28.2	−16.55	−14.76	2.61	76.05
**4**	−25	−13.0	−12.0	2.52	48.42
**6**(**a**)	−23.15	−13.0	−12.39	2.13	70.4
**5**	−22.07	−12.14	−11.24	1.82	48.3
**3**(**a**)	−21.81	−12.92	−13.03	2.22	59.42
**2**(**c**)	−20.71	−11.52	−10.92	2.16	59.42

**Table 3:** HBV inhibition in vitro measuring with the HepAD38 line, carrying the stably integrated HBV genome under the tetracycline-regulated promoter control (10 µM concentration of compounds **3**, **4** and **6**).

Mol	HBeAg ^1^ Inhibition, %	Cell Survival, %
**4**	83 ± 7%	91 ± 6%
**6**	79 ± 4%	78 ± 7%
**3**	68 ± 6%	87 ± 5%
zafirlukast (positive control)	82 ± 9%	89 ± 7%

^1^ HB-antigen of e-class virus replication-associated.

## Supplementary Materials

The ^1^H-NMR, ^13^C-NMR spectra of compounds and LCMS data are available online.
