# Supplementary material for: Methylation of Methyl 4-Hydroxy-2-thioxo-1,2-dihydroquinoline-3-carboxylate: Synthetic, Crystallographic, and Molecular Docking Studies
Source: Molecules. 2020 Sep 16;25(18):4238. doi: 10.3390/molecules25184238 (PMC7570464; doi:10.3390/molecules25184238)
Supplement: Supplementary file 1 [file molecules-25-04238-s001.pdf]

Figure S1. ^{1}H NMR spectrum (400 MHz, DMSO-d6) of methyl 4-hydroxy-2-(methylthio)quinoline-3-carboxylate **3**.
Figure S2. ^{13}C NMR spectrum (100 MHz, DMSO-d6) of methyl 4-hydroxy-2-(methylthio)quinoline-3-carboxylate **3**.
Figure S3. LC/MS data for methyl 4-hydroxy-2-(methylthio)quinoline-3-carboxylate **3**.
Figure S4. ^{1}H NMR spectrum (400 MHz, DMSO-d6) of pure methyl 4-methoxy-2-(methylthio)quinoline-3-carboxylate **4**.
Figure S5. ^{13}C NMR spectrum (100 MHz, DMSO-d6) of pure methyl 4-methoxy-2-(methylthio)quinoline-3-carboxylate **4**.
Figure S6. LC/MS data for pure methyl 4-methoxy-2-(methylthio)quinoline-3-carboxylate **4**.
Figure S7. ^{1}H NMR spectrum (400 MHz, DMSO-d6) of pure methyl 1-methyl-2-(methylthio)-4-oxo-1,4-dihydroquinoline-3-carboxylate **5**.
Figure S8. ^{13}C NMR spectrum (100 MHz, DMSO-d6) of pure methyl 1-methyl-2-(methylthio)-4-oxo-1,4-dihydroquinoline-3-carboxylate **5**.
Figure S9. LC/MS data for pure methyl 1-methyl-2-(methylthio)-4-oxo-1,4-dihydroquinoline-3-carboxylate **5**.
Figure S10. ^{1}H NMR spectrum (400 MHz, DMSO-d6) of the reaction product of case 1 (Table1).
Figure S11. LC/MS data for the reaction product of case 1 (Table1).
Figure S12. ^{1}H NMR spectrum (400 MHz, DMSO-d6) of the reaction product of case 2 (Table1).
Figure S13. LC/MS data for the reaction product of case 2 (Table1).
Figure S14. ^{1}H NMR spectrum (400 MHz, DMSO-d6) of the reaction product of case 3 (Table1).
Figure S15. LC/MS data for the reaction product of case 3 (Table1).
Figure S16. ^{1}H NMR spectrum (400 MHz, DMSO-d6) of the reaction product of case 4 (Table1).
Figure S17. LC/MS data for the reaction product of case 4 (Table1).
Figure S18. ^{1}H NMR spectrum (400 MHz, DMSO-d6) of the reaction product of case 5 (Table1).
Figure S19. LC/MS data for the reaction product of case 5 (Table1).
Figure S20. ^{1}H NMR spectrum (400 MHz, DMSO-d6) of the reaction product of case 6 (Table1).
Figure S21. LC/MS data for the reaction product of case 6 (Table1).
Figure S22. ^{1}H NMR spectrum (400 MHz, DMSO-d6) of the reaction product of case 7 (Table1).
Figure S23. LC/MS data for the reaction product of case 7 (Table1).
Figure S24. ^{1}H NMR spectrum (400 MHz, DMSO-d6) of the reaction product of case 8 (Table1).
Figure S25. LC/MS data for the reaction product of case 8 (Table1).
Figure S26. ^{1}H NMR spectrum (400 MHz, DMSO-d6) of the reaction product of case 9 (Table1).
Figure S27. LC/MS data for the reaction product of case 9 (Table1).
Figure S28. ^{1}H NMR spectrum (400 MHz, DMSO-d6) of the reaction product of case 10 (Table1).
Figure S29. LC/MS data for the reaction product of case 10 (Table1).
Figure S30. ^{1}H NMR spectrum (400 MHz, DMSO-d6) of 4-hydroxy-2-(methylthio)quinoline-3-carboxylic acid **6**.
Figure S31. ^{13}C NMR spectrum (100 MHz, DMSO-d6) of 4-hydroxy-2-(methylthio)quinoline-3-carboxylic acid **6**.
Figure S32. LC/MS data for 4-hydroxy-2-(methylthio)quinoline-3-carboxylic acid **6**.

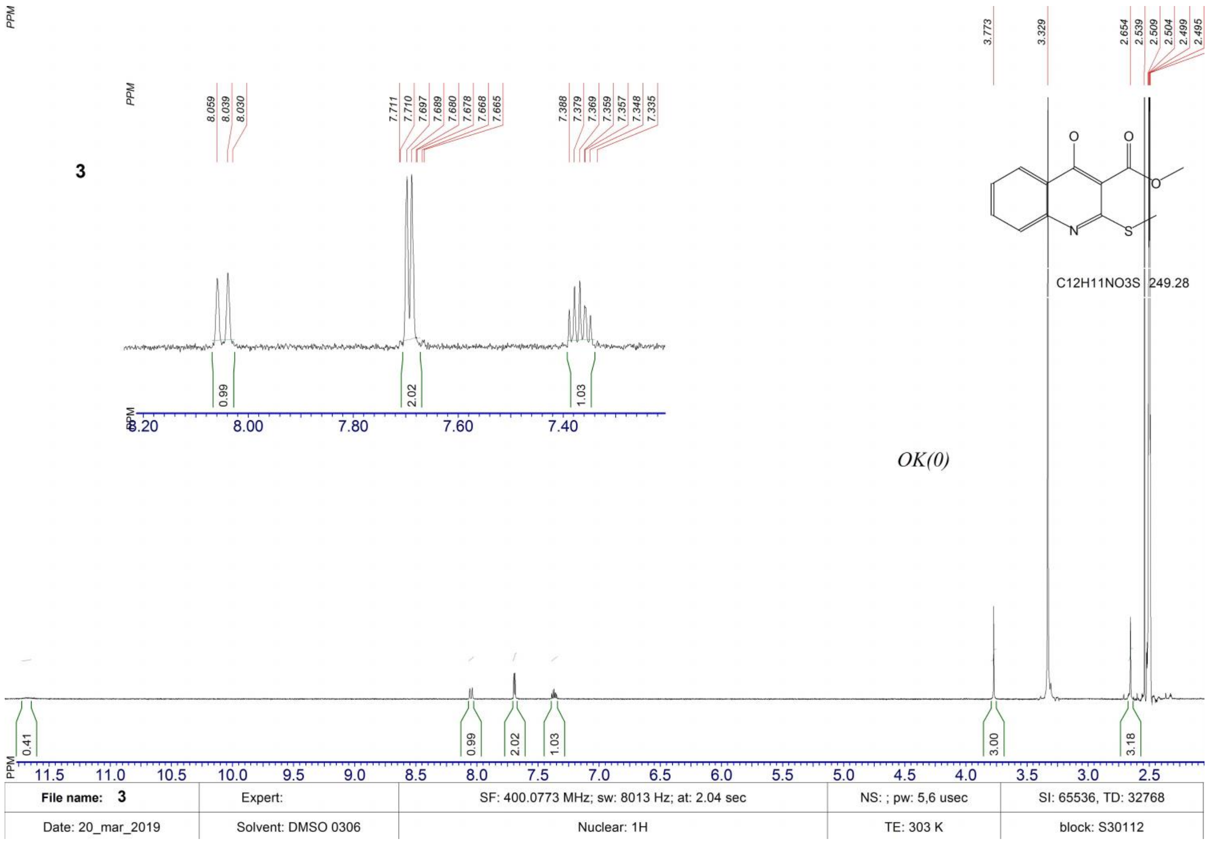

Figure S1. ^{1}H NMR spectrum (400 MHz, DMSO-d6) of methyl 4-hydroxy-2-(methylthio)quinoline-3-carboxylate **3**.

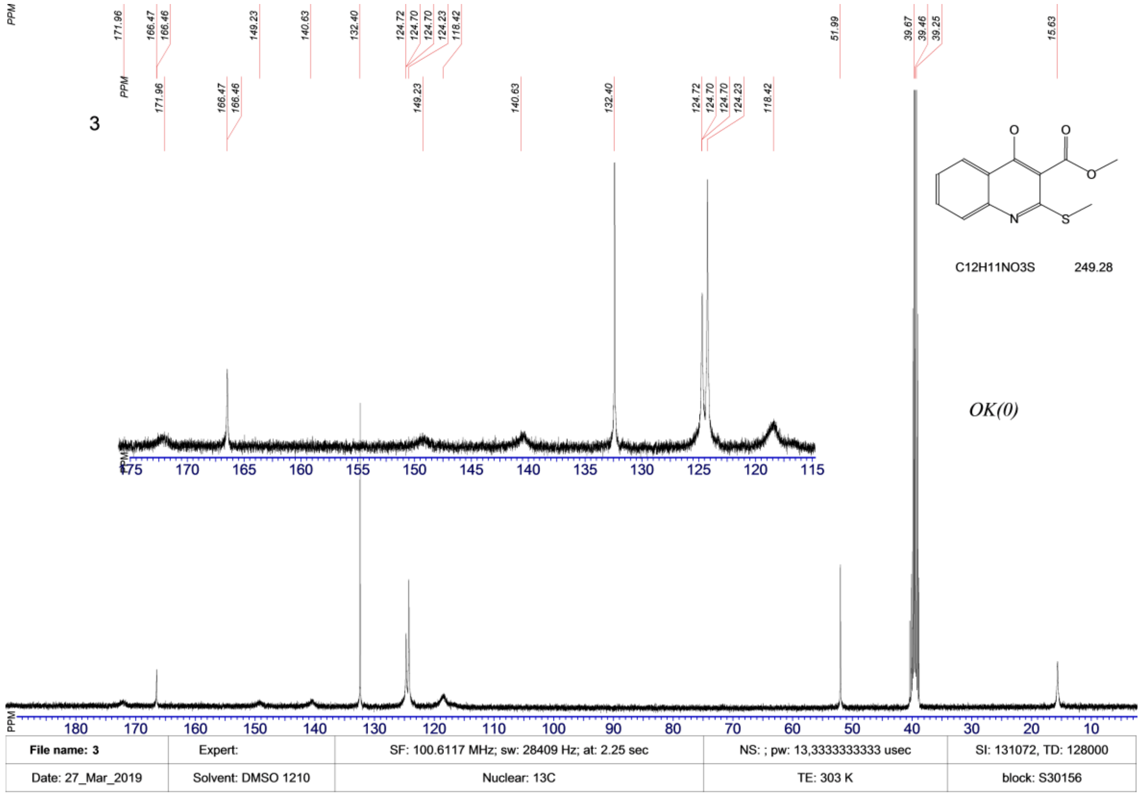

Figure S2. ^{13}C NMR spectrum (100 MHz, DMSO-d6) of methyl 4-hydroxy-2-(methylthio)quinoline-3-carboxylate **3**.

-.o.-Syntez Purity Report -.o.-

Agilent 1200 LC/MSD
Diodearrey G1315B (DAD1A-215nm; DAD1B-241nm)
Mass Quad G6140A (MSD1-Pos, MSD2-Neg)
ELSD Altech 3300 (ADC1 A, ELSD)

Mobile Phase:A-H2O+0.1HCOOH;B-MeCN+0.1HCOOH
Separation column: **99%**
Rapid Resolutionn HT Cartige 4.6x30mm,
1.8-Micron, Zorbax SB-C18

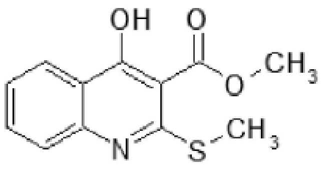

Mw 249,29

M30453 ->

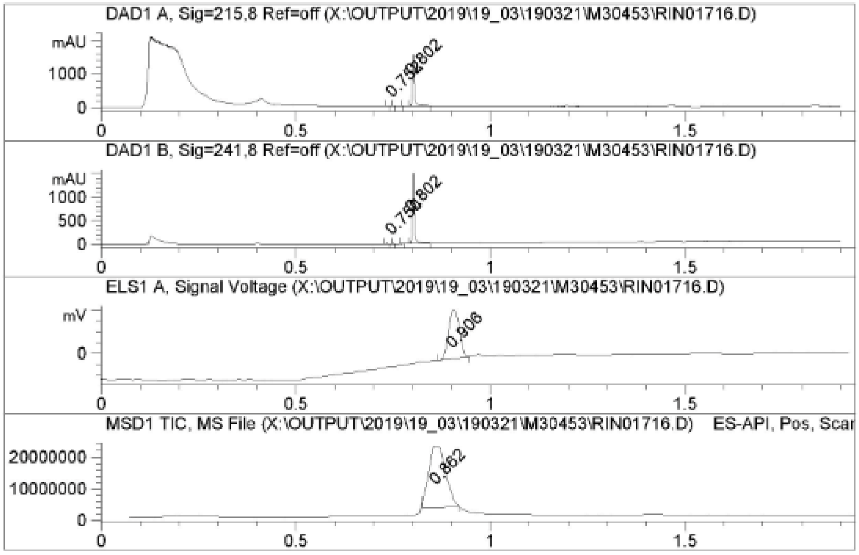

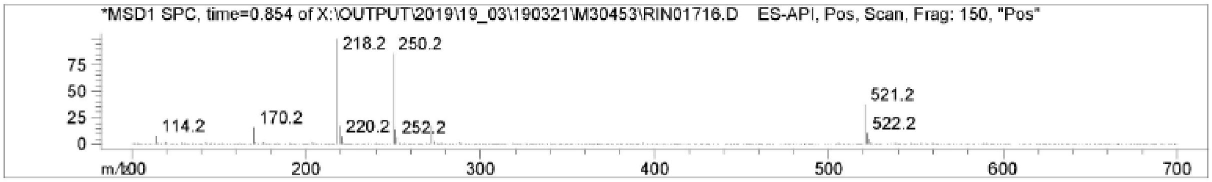

#	Signal	R.Time	Area %
1	DAD1 A, Sig=215,8 Ref=off	0.733	0.251
2		0.752	0.216
3		0.773	0.180
4		0.802	99.353

#	Signal	R.Time	Area %
1	DAD1 B, Sig=241,8 Ref=off	0.734	0.312
2		0.750	0.145
3		0.773	0.225
4		0.802	99.318

#	Signal	R.Time	Area %
1	ELS1 A, Signal Voltage	0.906	100.000

#	Signal	R.Time	Area %
1	MSD1 TIC, MS File	0.862	100.000

Figure S3. LC/MS data for methyl 4-hydroxy-2-(methylthio)quinoline-3-carboxylate **3**.

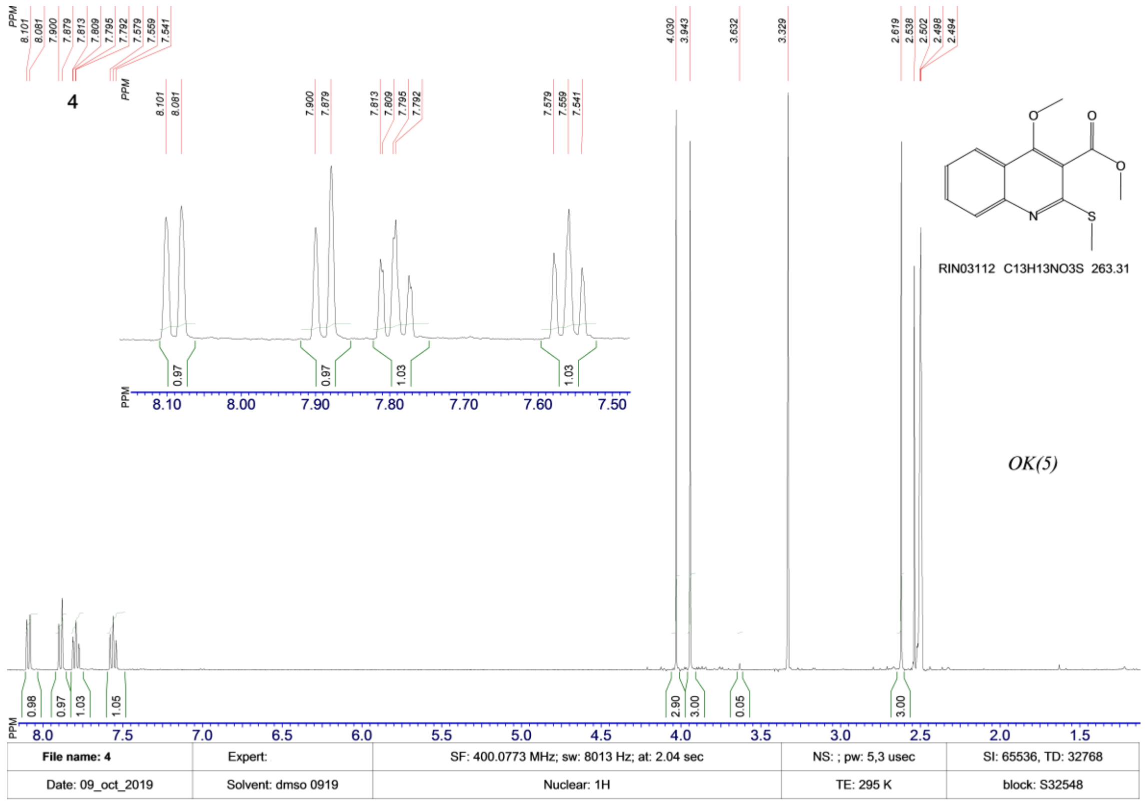

Figure S4. ^{1}H NMR spectrum (400 MHz, DMSO-d6) of pure methyl 4-methoxy-2-(methylthio)quinoline-3-carboxylate **4**.

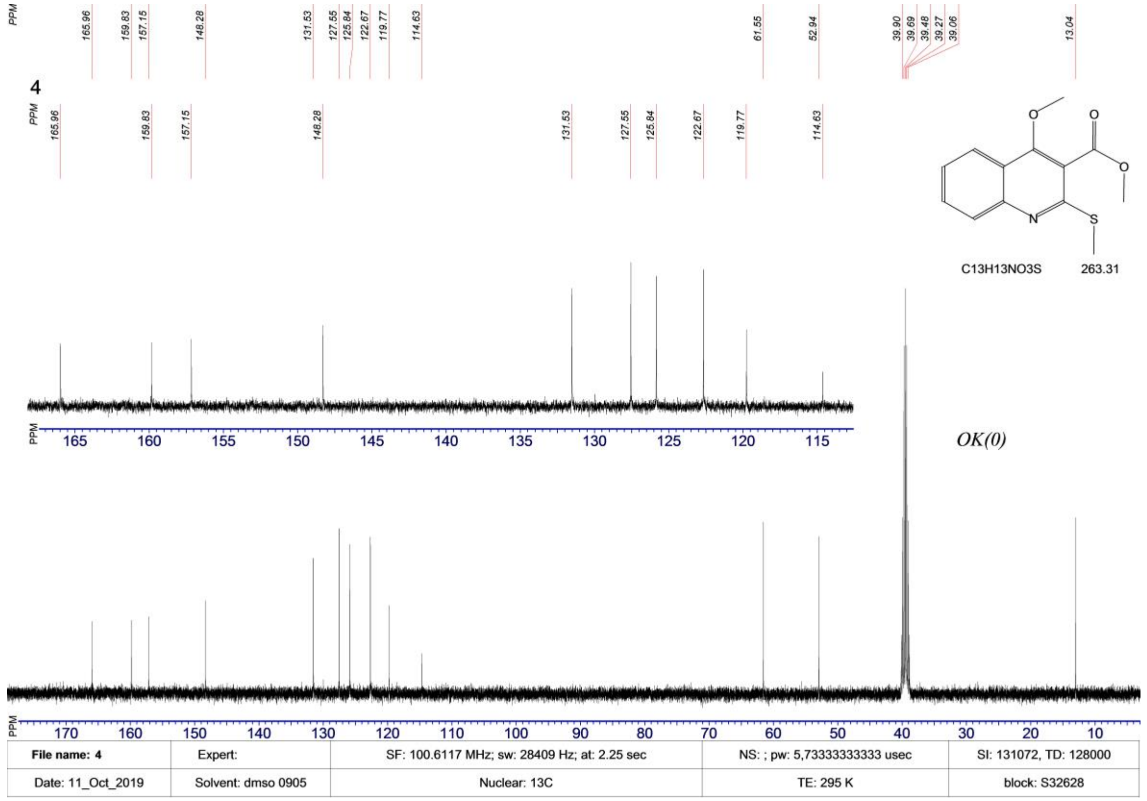

Figure S5. ^{13}C NMR spectrum (100 MHz, DMSO-d6) of pure methyl 4-methoxy-2-(methylthio)quinoline-3-carboxylate **4**.

-.o.-Syntez Purity Report -.o.-

Agilent 1200 LC/MSD
Diodearrey G1315B (DAD1A-215nm; DAD1B-241nm)
Mass Quad G6140A (MSD1-Pos, MSD2-Neg)
ELSD Altech 3300 (ADC1 A, ELSD)

Mobile Phase:A-H2O+0.1HCOOH;B-MeCN+0.1HCOOH
Separation column: **96%**
Rapid Resolutionn HT Cartige 4.6x30mm,
1.8-Micron, Zorbax SB-C18

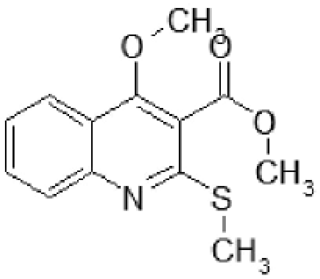

Mw 263,32

M32531 ->

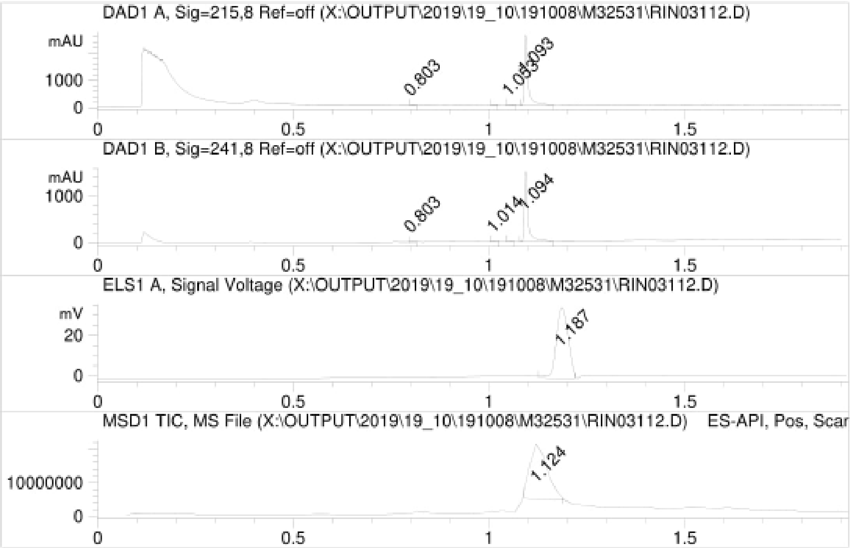

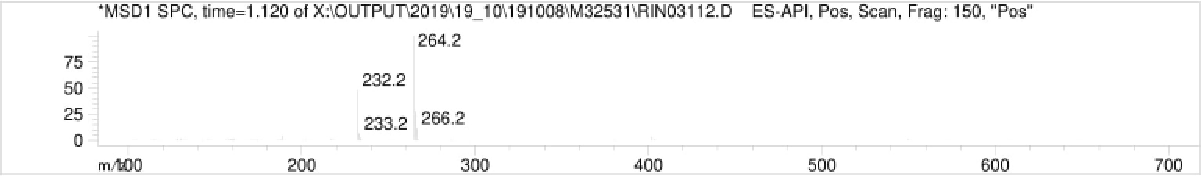

#	Signal	R.Time	Area %
1	DAD1 A, Sig=215,8 Ref=off	0.803	0.805
2		1.014	0.809
3		1.053	0.717
4		1.093	97.668

#	Signal	R.Time	Area %
1	DAD1 B, Sig=241,8 Ref=off	0.803	0.897
2		1.014	1.152
3		1.053	1.705
4		1.094	96.246

#	Signal	R.Time	Area %
1	ELS1 A, Signal Voltage	1.187	100.000

#	Signal	R.Time	Area %
1	MSD1 TIC, MS File	1.124	100.000

Figure S6. LC/MS data for pure methyl 4-methoxy-2-(methylthio)quinoline-3-carboxylate **4**.

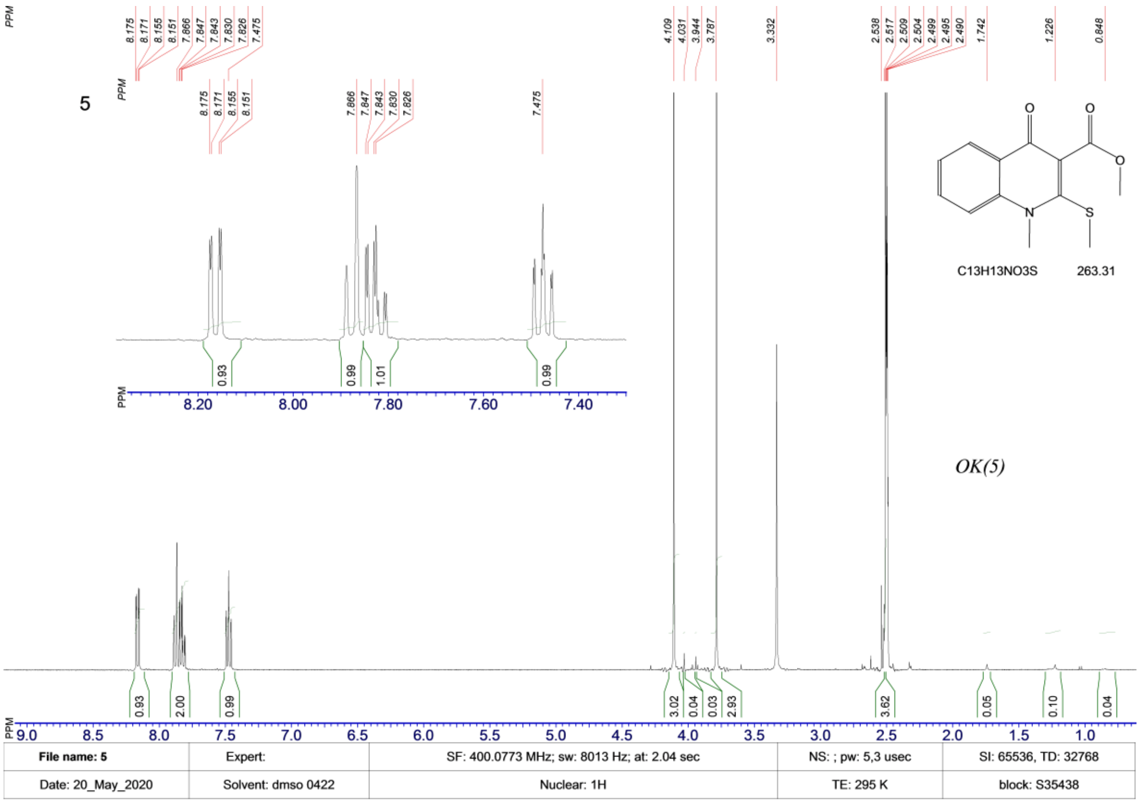

Figure S7. ^{1}H NMR spectrum (400 MHz, DMSO-d6) of pure methyl 1-methyl-2-(methylthio)-4-oxo-1,4-dihydroquinoline-3-carboxylate **5**.

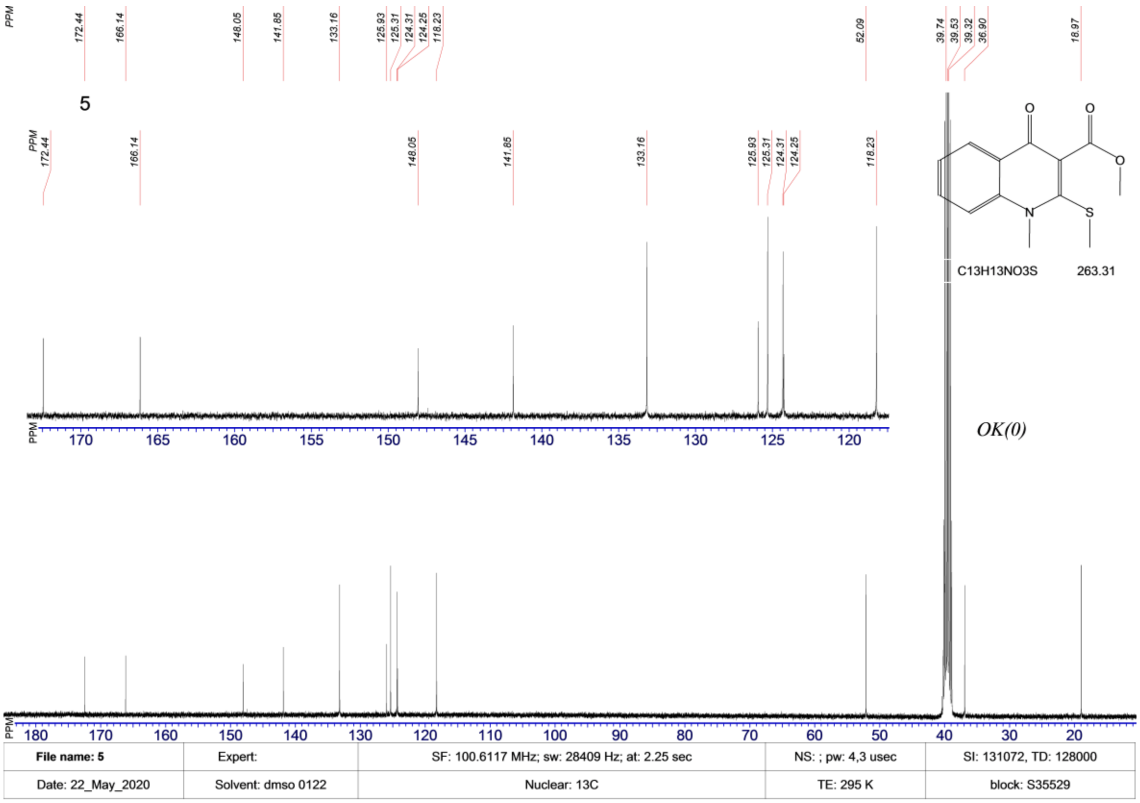

Figure S8. ^{13}C NMR spectrum (100 MHz, DMSO-d6) of pure methyl 1-methyl-2-(methylthio)-4-oxo-1,4-dihydroquinoline-3-carboxylate **5**.

```
                    -.o.-Syntez Purity Report -.o.-
Agilent 1100 LC/MSD                                     Mobile Phase:A-H2O+0.1HCOOH;B-MeCN+0.1HCOOH
Diodearrey G1315B (DAD1A-215nm; DAD1B-240nm)            Separation column:
Mass Quad  G1956B  (MSD1-Pos, MSD2-Neg)                 Rapid Resolutionn HT Cartige 4.6x30mm,
ELSD Altech 3300  (ADC1 A, ELSD)                        1.8-Micron, Zorbax SB-C18
------------------------------------------------------------------------------------------------->
```

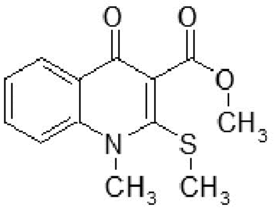

Mw 263,32

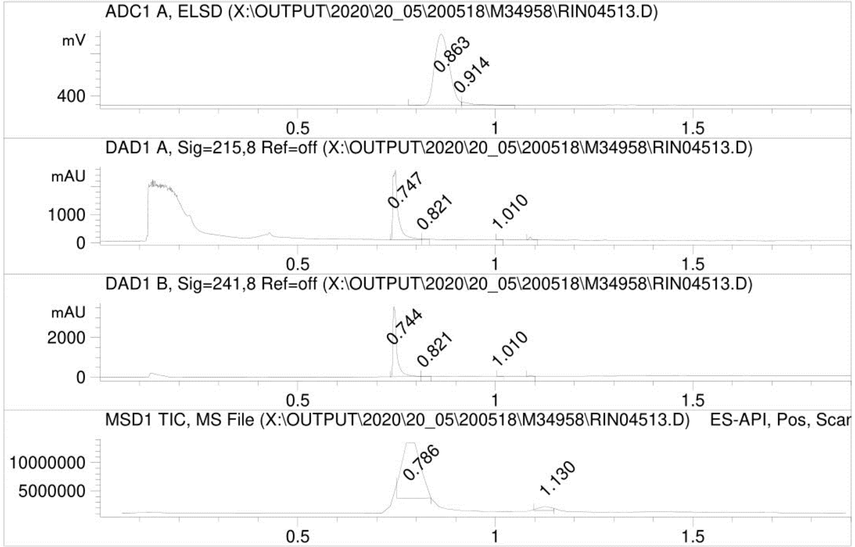


```
------------------------------------------------------------------------------------------------->
```

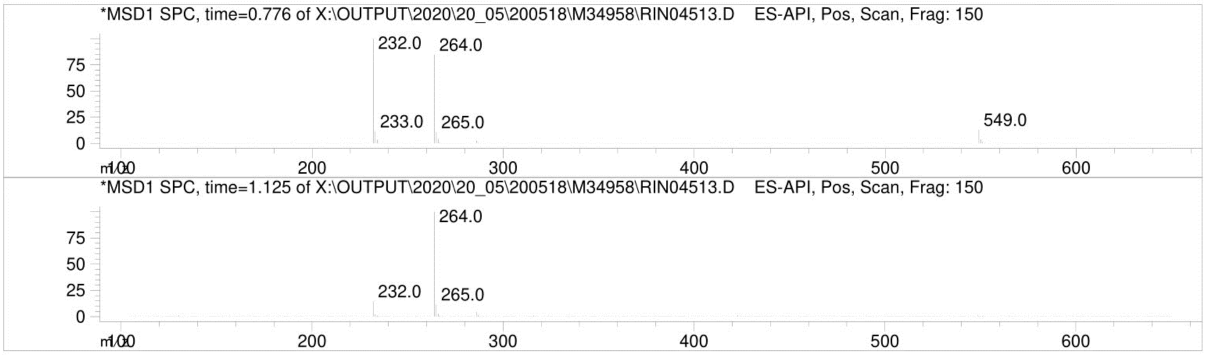

#	Signal	R.Time	Area %
1	ADC1 A, ELSD	0.863	97.326
2		0.914	2.674

#	Signal	R.Time	Area %
1	DAD1 A, Sig=215,8 Ref=off	0.747	97.015
2		0.821	0.701
3		1.010	0.281
4		1.088	2.003

#	Signal	R.Time	Area %
1	DAD1 B, Sig=241,8 Ref=off	0.744	97.694
2		0.821	0.674
3		1.010	0.491
4		1.088	1.141

#	Signal	R.Time	Area %
1	MSD1 TIC, MS File	0.786	94.739
2		1.130	5.261

Figure S9. LC/MS data for pure methyl 1-methyl-2-(methylthio)-4-oxo-1,4-dihydroquinoline-3-carboxylate **5**.

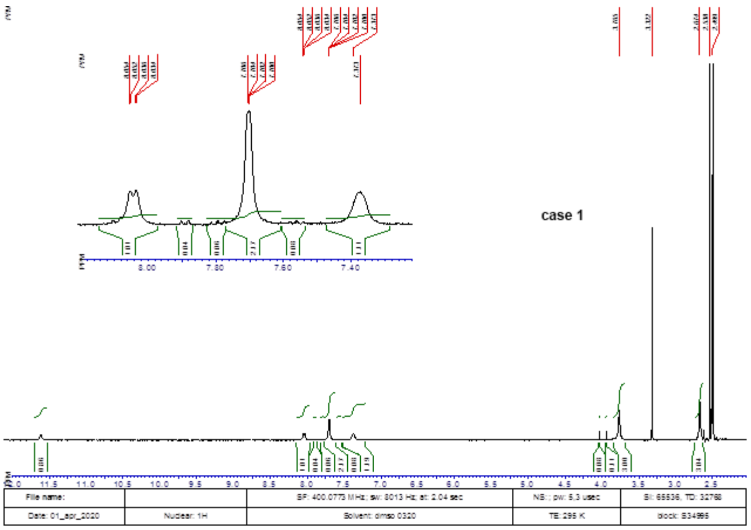

Figure S10. ^{1}H NMR spectrum (400 MHz, DMSO-d6) of the reaction product of case 1 (Table 1).

-.o.-Syntez Purity Report -.o.-

Agilent 1100 LC/MSD	Mobile Phase:A-H2O+0.1HCOOH;B-MeCN+0.1HCOOH
Diodearrey G1315B (DAD1A-215nm; DAD1B-240nm)	Separation column:
Mass Quad G1956B (MSD1-Pos, MSD2-Neg)	Rapid Resolutionn HT Cartige 4.6x30mm,
ELSD Altech 3300 (ADC1 A, ELSD)	1.8-Micron, Zorbax SB-C18

case 1

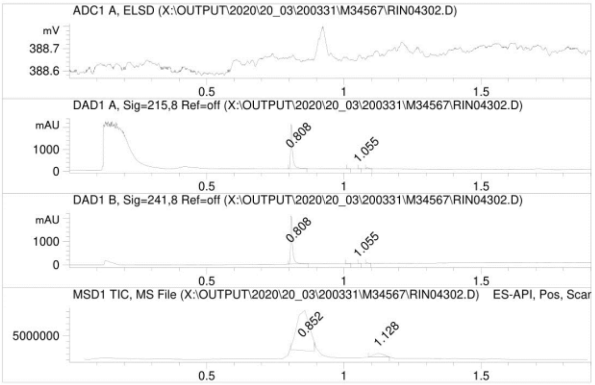

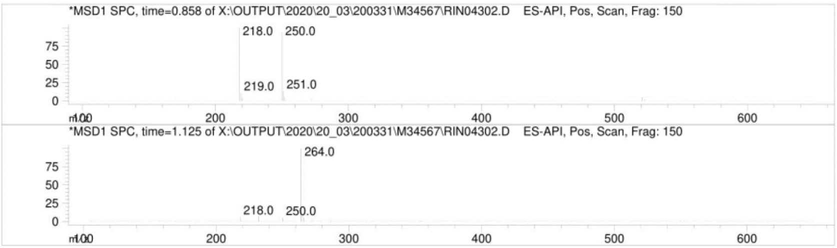

#	Signal	R.Time	Area %

#	Signal	R.Time	Area %
1	DAD1 A, Sig=215,8 Ref=off	0.808	96.191
2		1.014	0.399
3		1.055	0.331
4		1.086	3.079

#	Signal	R.Time	Area %
1	DAD1 B, Sig=241,8 Ref=off	0.808	97.406
2		1.014	0.768
3		1.055	0.265
4		1.086	1.561

#	Signal	R.Time	Area %
1	MSD1 TIC, MS File	0.852	93.840
2		1.128	6.160

Figure S11. LC/MS data for the reaction product of case 1 (Table 1).

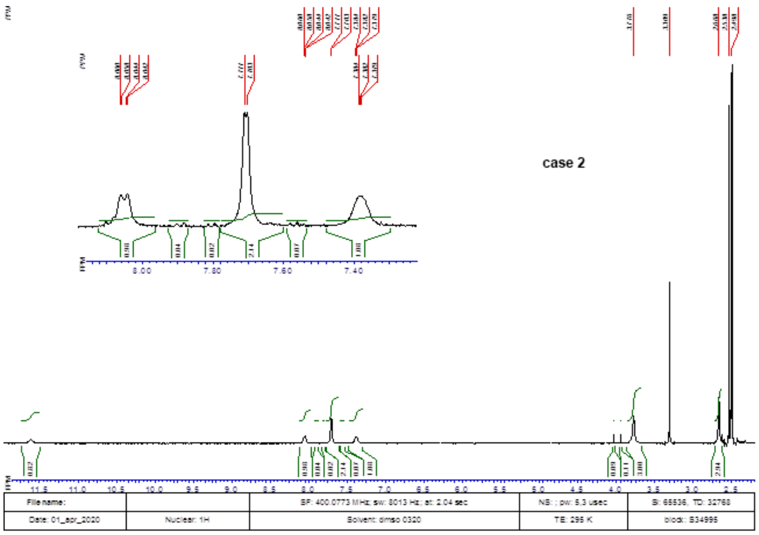

Figure S12. ^{1}H NMR spectrum (400 MHz, DMSO-d6) of the reaction product of case 2 (Table 1).

```
                         -.o.-Syntez Purity Report -.o.-
Agilent 1100 LC/MSD                               Mobile Phase:A-H2O+0.1HCOOH;B-MeCN+0.1HCOOH
Diodearrey G1315B (DAD1A-215nm; DAD1B-240nm)      Separation column:
Mass Quad  G1956B  (MSD1-Pos, MSD2-Neg)           Rapid Resolutionn HT Cartige 4.6x30mm,
ELSD Altech 3300  (ADC1 A, ELSD)                  1.8-Micron, Zorbax SB-C18
```

case 2

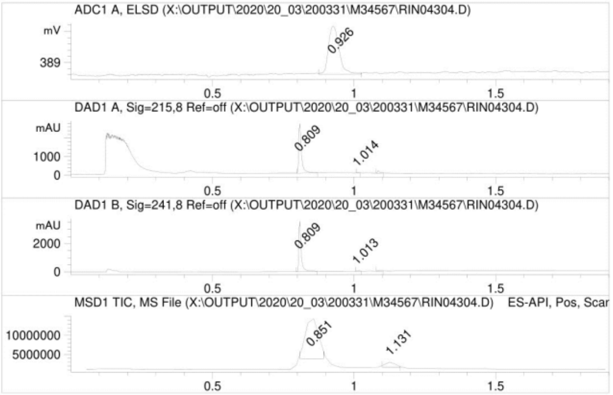

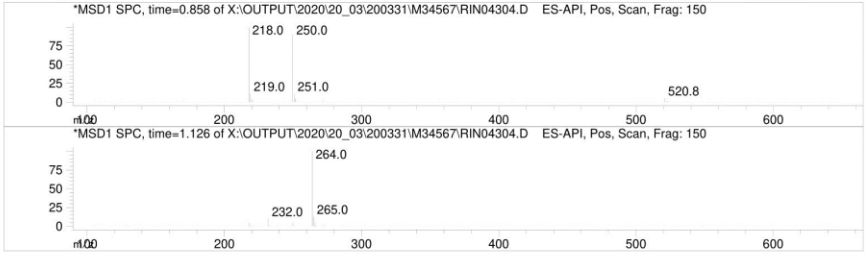

#	Signal	R.Time	Area %
1	ADC1 A, ELSD	0.926	100.000

#	Signal	R.Time	Area %
1	DAD1 A, Sig=215,8 Ref=off	0.809	96.060
2		1.014	0.353
3		1.086	3.587

#	Signal	R.Time	Area %
1	DAD1 B, Sig=241,8 Ref=off	0.809	97.616
2		1.013	0.577
3		1.086	1.807

#	Signal	R.Time	Area %
1	MSD1 TIC, MS File	0.851	92.305
2		1.131	7.695

Figure S13. LC/MS data for the reaction product of case 2 (Table 1).

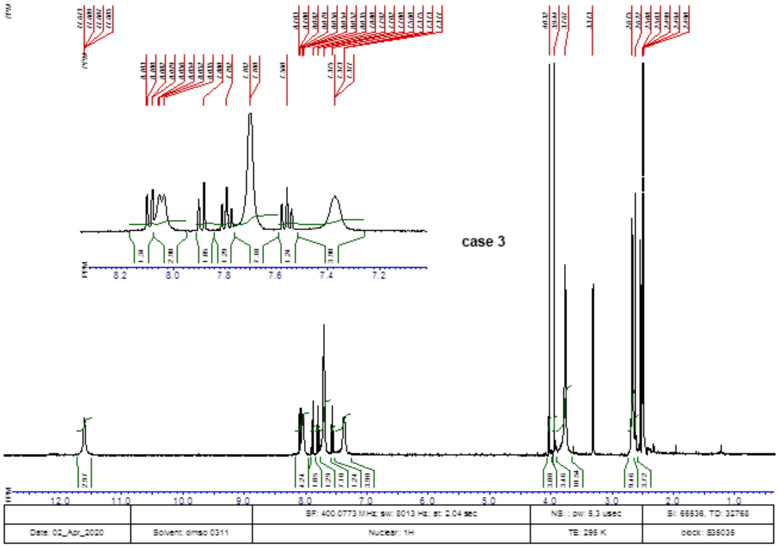

Figure S14. ^{1}H NMR spectrum (400 MHz, DMSO-d6) of the reaction product of case 3 (Table 1).

```
                    -.o.-Syntez Purity Report -.o.-
Agilent 1100 LC/MSD                                   Mobile Phase:A-H2O+0.1HCOOH;B-MeCN+0.1HCOOH
Diodearrey G1315B (DAD1A-215nm; DAD1B-240nm)          Separation column:
Mass Quad  G1956B  (MSD1-Pos, MSD2-Neg)               Rapid Resolutionn HT Cartige 4.6x30mm,
ELSD Altech 3300  (ADC1 A, ELSD)                      1.8-Micron, Zorbax SB-C18
```

case 3

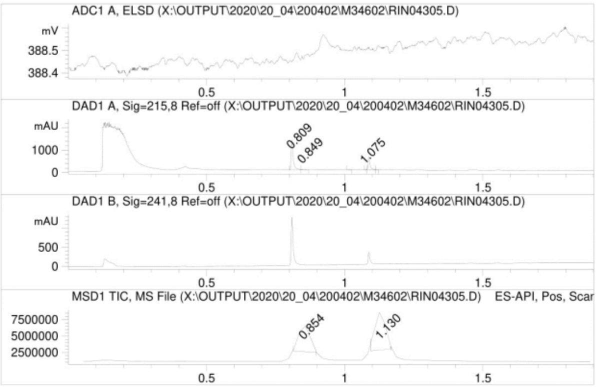

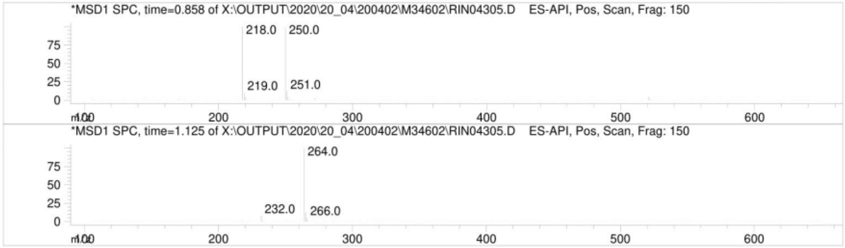

#	Signal	R.Time	Area %

#	Signal	R.Time	Area %
1	DAD1 A, Sig=215,8 Ref=off	0.809	68.939
2		0.849	1.345
3		1.014	0.480
4		1.075	0.093
5		1.088	28.872
6		1.111	0.272

#	Signal	R.Time	Area %

#	Signal	R.Time	Area %
1	MSD1 TIC, MS File	0.854	44.592
2		1.130	55.408

Figure S15. LC/MS data for the reaction product of case 3 (Table 1).

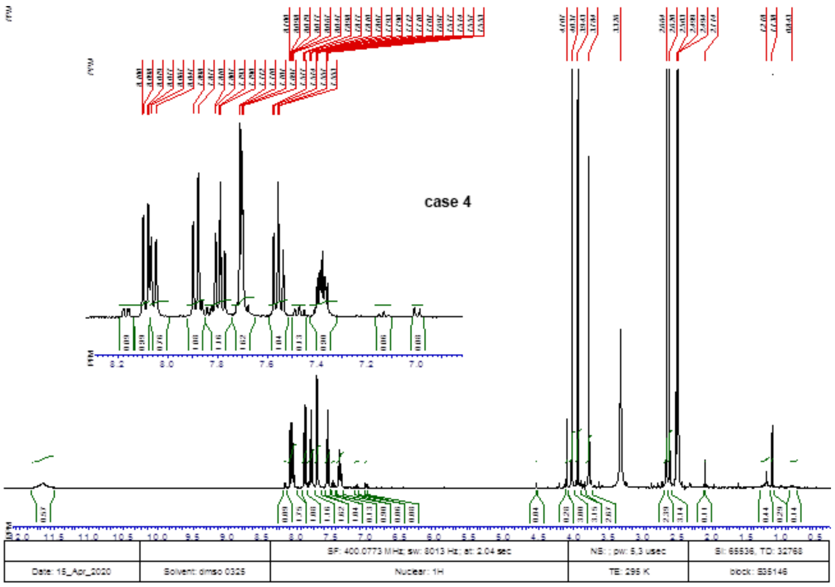

Figure S16. ^{1}H NMR spectrum (400 MHz, DMSO-d6) of the reaction product of case 4 (Table 1).

```
                    -.o.-Syntez Purity Report -.o.-
Agilent 1200 LC/MSD                              Mobile Phase:A-H2O+0.1HCOOH;B-MeCN+0.1HCOOH
Diodearrey G1315B (DAD1A-215nm; DAD1B-241nm)     Separation column:
Mass Quad  G6140A (MSD1-Pos,   MSD2-Neg)         Rapid Resolutionn HT Cartige 4.6x30mm,
ELSD Altech 3300  (ADC1 A, ELSD)                 1.8-Micron, Zorbax SB-C18
------------------------------------------------------------------------------------->
```

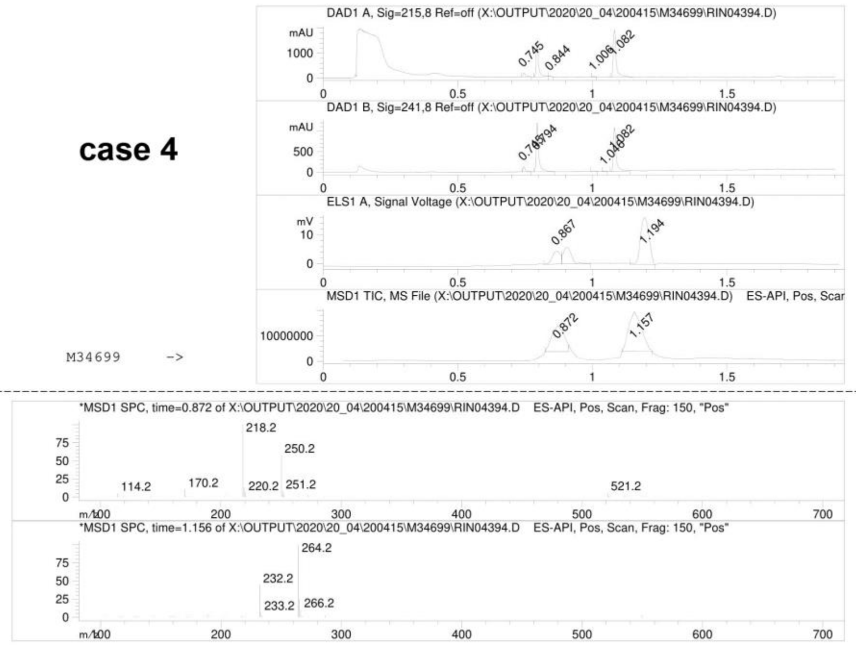

#	Signal	R.Time	Area %
1	DAD1 A, Sig=215,8 Ref=off	0.745	4.213
2		0.794	34.183
3		0.844	0.151
4		1.006	0.391
5		1.082	61.062

#	Signal	R.Time	Area %
1	DAD1 B, Sig=241,8 Ref=off	0.745	4.701
2		0.794	48.401
3		1.005	0.641
4		1.046	0.211
5		1.082	46.046

#	Signal	R.Time	Area %
1	ELS1 A, Signal Voltage	0.867	16.012
2		0.905	21.208
3		1.194	62.779

Figure S17. LC/MS data for the reaction product of case 4 (Table 1).

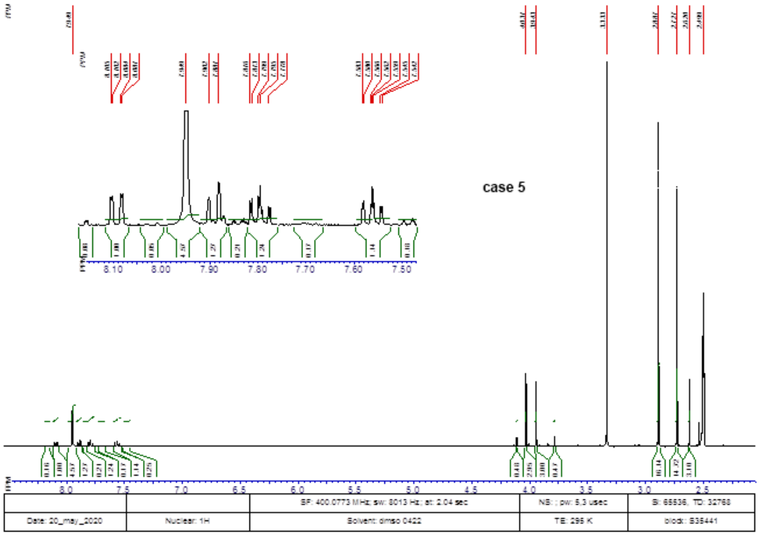

Figure S18. ^{1}H NMR spectrum (400 MHz, DMSO-d6) of the reaction product of case 5 (Table 1).

```
                         -.o.-Syntez Purity Report -.o.-
Agilent 1200 LC/MSD                                  Mobile Phase:A-H2O+0.1HCOOH;B-MeCN+0.1HCOOH
Diodearrey G1315B (DAD1A-215nm; DAD1B-241nm)         Separation column:
Mass Quad  G6140A (MSD1-Pos,   MSD2-Neg)             Rapid Resolutionn HT Cartige 4.6x30mm,
ELSD Altech 3300  (ADC1 A, ELSD)                     1.8-Micron, Zorbax SB-C18
```

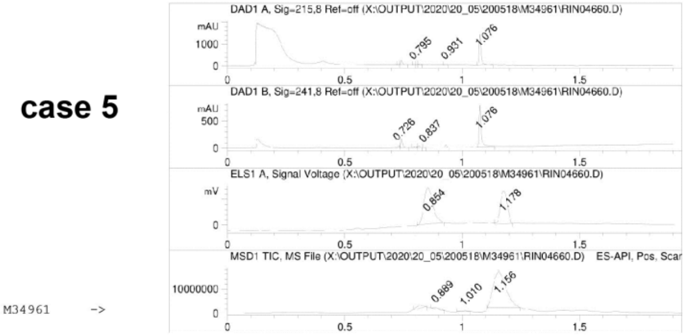

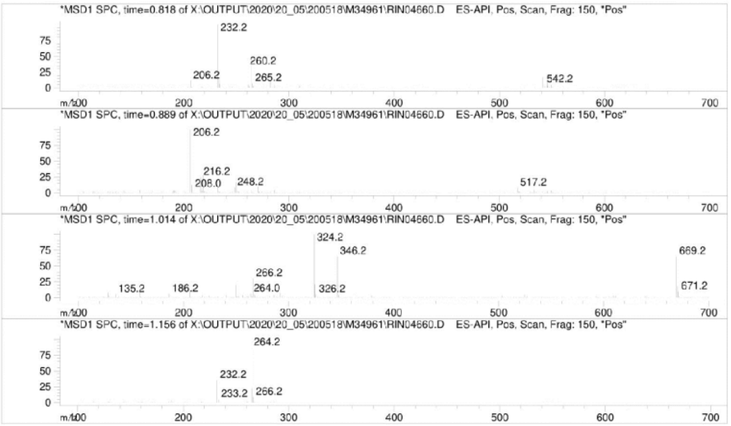

#	Signal	R.Time	Area %
1	DAD1 A, Sig=215,8 Ref=off	0.728	1.091
2		0.742	11.612
3		0.795	0.849
4		0.806	1.251
5		0.816	2.674
6		0.931	1.223
7		1.076	81.299

#	Signal	R.Time	Area %
1	DAD1 B, Sig=241,8 Ref=off	0.726	3.202
2		0.741	15.434
3		0.794	1.011
4		0.805	1.303
5		0.816	3.288
6		0.837	0.393
7		0.930	3.455
8		1.076	71.913

Figure S19. LC/MS data for the reaction product of case 5 (Table 1).

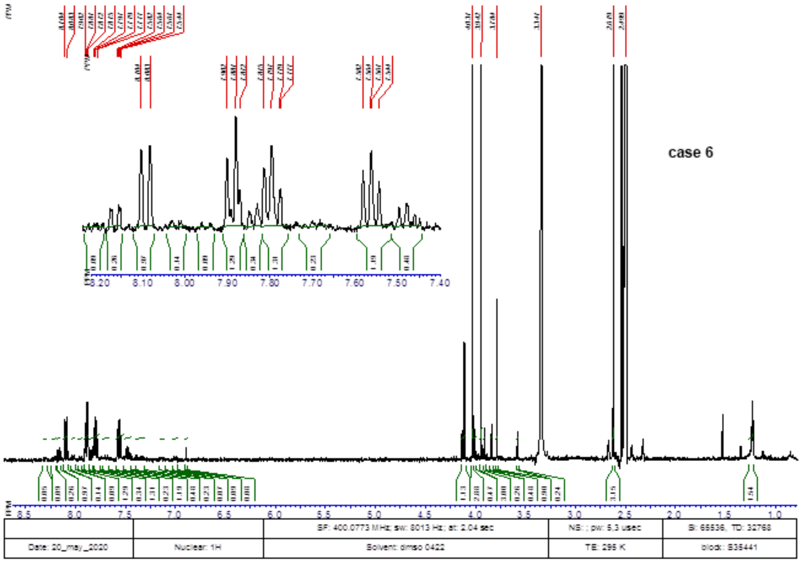

Figure S20. ^{1}H NMR spectrum (400 MHz, DMSO-d6) of the reaction product of case 6 (Table 1).

```
-.o.-Syntez Purity Report -.o.-
Agilent 1200 LC/MSD                              Mobile Phase:A-H2O+0.1HCOOH;B-MeCN+0.1HCOOH
Diodearrey G1315B (DAD1A-215nm; DAD1B-241nm)     Separation column:
Mass Quad  G6140A (MSD1-Pos,    MSD2-Neg)        Rapid Resolutionn HT Cartige 4.6x30mm,
ELSD Altech 3300  (ADC1 A, ELSD)                 1.8-Micron, Zorbax SB-C18
```

case 6

M34961 ->

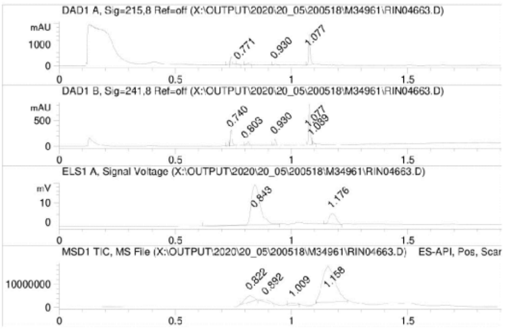

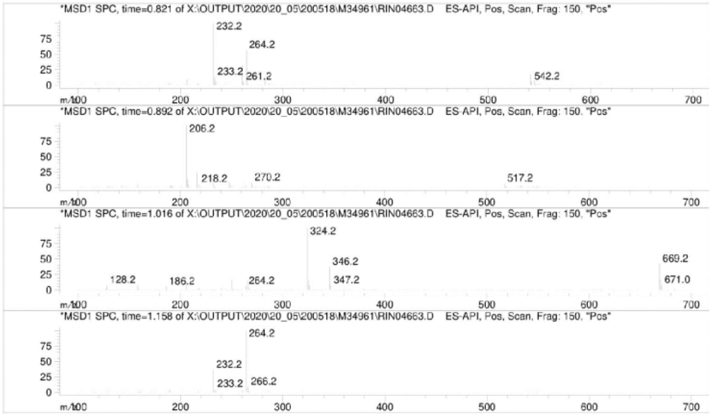

#	Signal	R.Time	Area %
1	DAD1 A, Sig=215,8 Ref=off	0.725	1.653
2		0.740	17.306
3		0.771	0.998
4		0.802	2.526
5		0.814	4.265
6		0.930	3.580
7		1.077	69.672

#	Signal	R.Time	Area %
1	DAD1 B, Sig=241,8 Ref=off	0.725	4.975
2		0.740	21.937
3		0.803	2.739
4		0.814	4.791
5		0.930	8.656
6		1.077	53.705
7		1.089	3.197

Figure S21. LC/MS data for the reaction product of case 6 (Table 1).

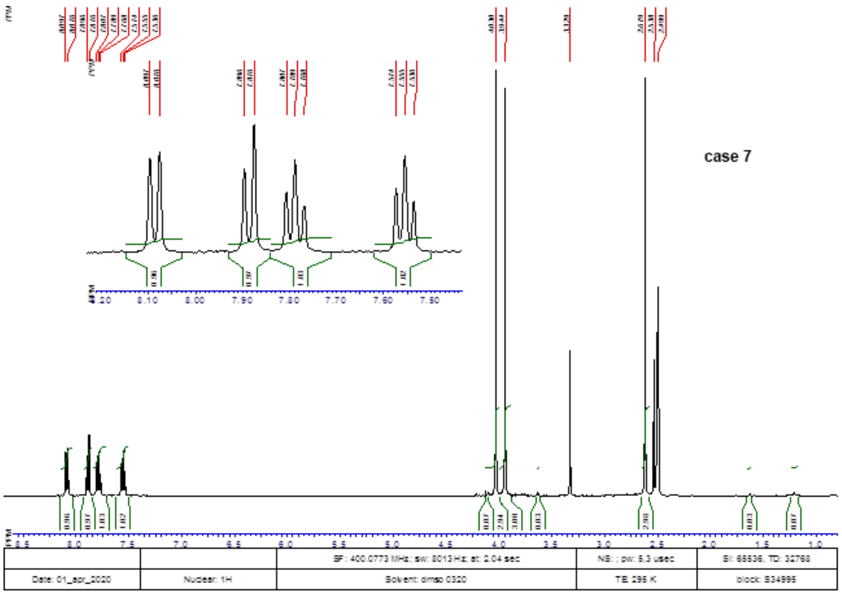

Figure S22. ^{1}H NMR spectrum (400 MHz, DMSO-d6) of the reaction product of case 7 (Table 1).

-.o.-Syntez Purity Report -.o.-

Agilent 1100 LC/MSD
Diodearrey G1315B (DAD1A-215nm; DAD1B-240nm)
Mass Quad G1956B (MSD1-Pos, MSD2-Neg)
ELSD Altech 3300 (ADC1 A, ELSD)

Mobile Phase:A-H2O+0.1HCOOH;B-MeCN+0.1HCOOH
Separation column: **96%**
Rapid Resolutionn HT Cartige 4.6x30mm,
1.8-Micron, Zorbax SB-C18

case 7

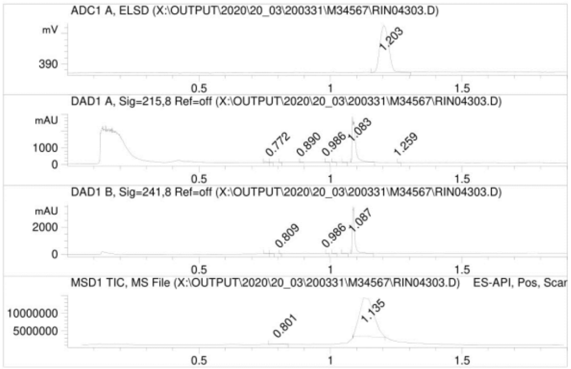

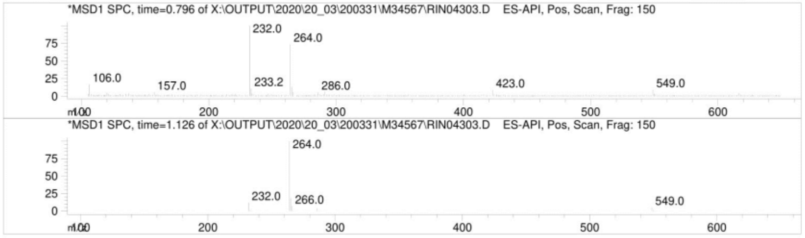

#	Signal	R.Time	Area %
1	ADC1 A, ELSD	1.203	100.000

#	Signal	R.Time	Area %
1	DAD1 A, Sig=215,8 Ref=off	0.752	1.068
2		0.772	0.142
3		0.809	0.101
4		0.890	0.088
5		0.986	0.385
6		1.012	0.654
7		1.050	0.705
8		1.083	96.741
9		1.259	0.116

#	Signal	R.Time	Area %
1	DAD1 B, Sig=241,8 Ref=off	0.752	0.943
2		0.773	0.311
3		0.809	0.084
4		0.986	0.226
5		1.012	0.566
6		1.051	0.913
7		1.087	96.957

Figure S23. LC/MS data for the reaction product of case 7 (Table 1).

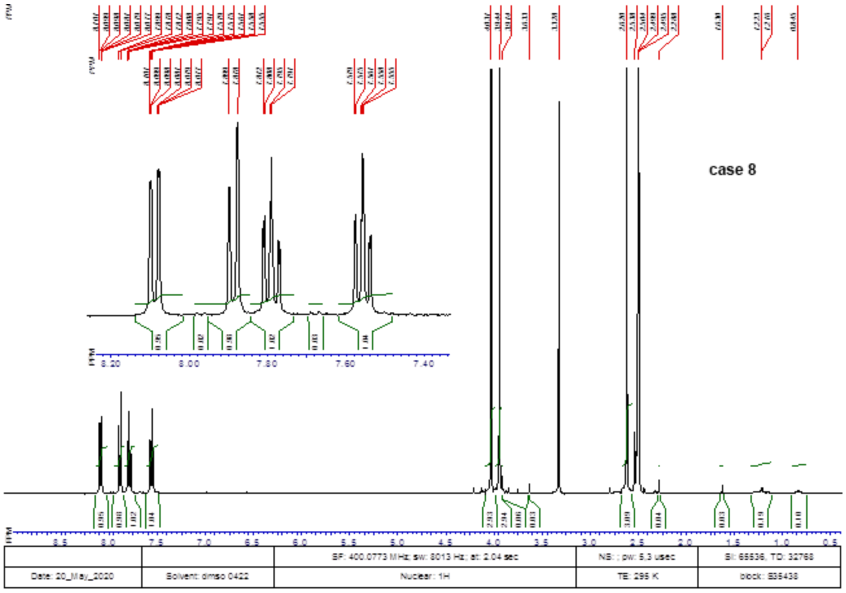

Figure S24. ^{1}H NMR spectrum (400 MHz, DMSO-d6) of the reaction product of case 8 (Table 1).

```
                     -.o.-Syntez Purity Report -.o.-
Agilent 1100 LC/MSD                            Mobile Phase:A-H2O+0.1HCOOH;B-MeCN+0.1HCOOH
Diodearrey G1315B (DAD1A-215nm; DAD1B-240nm)   Separation column: 96%
Mass Quad  G1956B  (MSD1-Pos, MSD2-Neg)        Rapid Resolutionn HT Cartige 4.6x30mm,
ELSD Altech 3300  (ADC1 A, ELSD)               1.8-Micron, Zorbax SB-C18
```

case 8

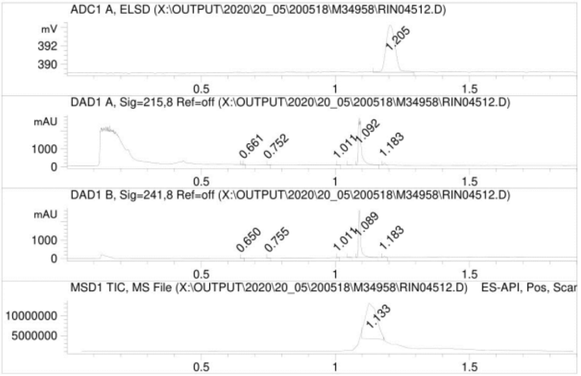

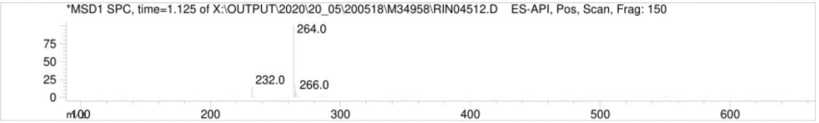

#	Signal	R.Time	Area %
1	ADC1 A, ELSD	1.205	100.000

#	Signal	R.Time	Area %
1	DAD1 A, Sig=215,8 Ref=off	0.650	0.102
2		0.661	0.063
3		0.752	0.182
4		1.011	0.333
5		1.052	0.459
6		1.092	96.351
7		1.183	2.511

#	Signal	R.Time	Area %
1	DAD1 B, Sig=241,8 Ref=off	0.650	0.194
2		0.755	0.110
3		1.011	0.264
4		1.052	0.908
5		1.089	96.891
6		1.183	1.633

#	Signal	R.Time	Area %
1	MSD1 TIC, MS File	1.133	100.000

Figure S25. LC/MS data for the reaction product of case 8 (Table 1).

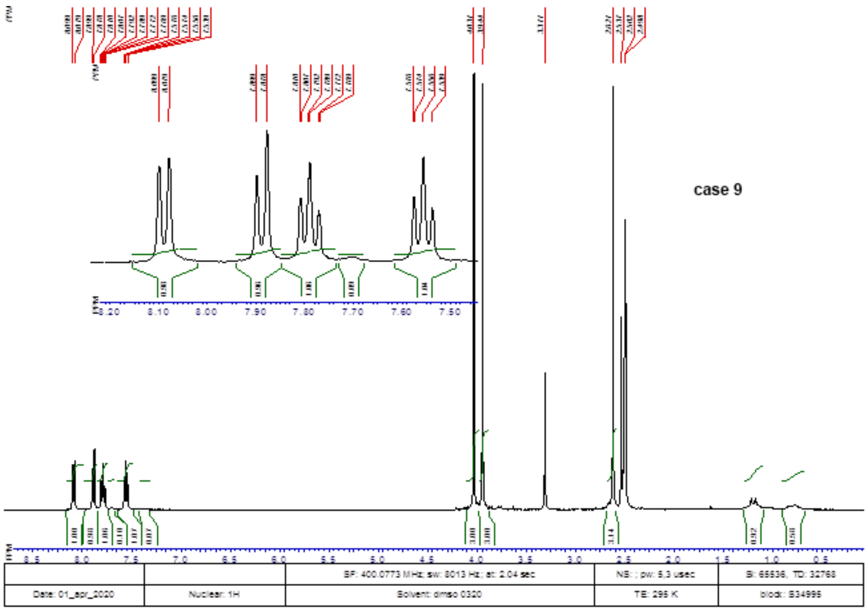

Figure S26. ^{1}H NMR spectrum (400 MHz, DMSO-d6) of the reaction product of case 9 (Table 1).

```
-.o.-Syntez Purity Report -.o.-
Agilent 1100 LC/MSD                         Mobile Phase:A-H2O+0.1HCOOH;B-MeCN+0.1HCOOH
Diodearrey G1315B (DAD1A-215nm; DAD1B-240nm) Separation column: 95%
Mass Quad  G1956B  (MSD1-Pos, MSD2-Neg)      Rapid Resolutionn HT Cartige 4.6x30mm,
ELSD Altech 3300  (ADC1 A, ELSD)             1.8-Micron, Zorbax SB-C18
```

case 9

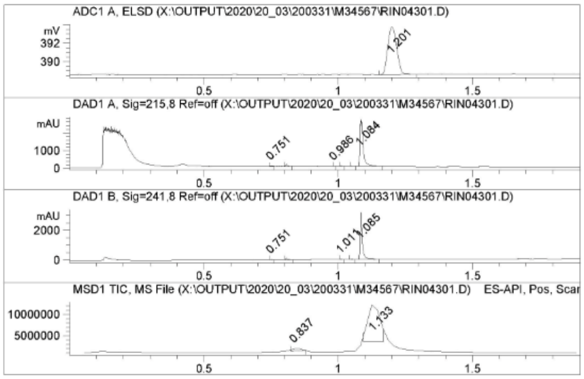

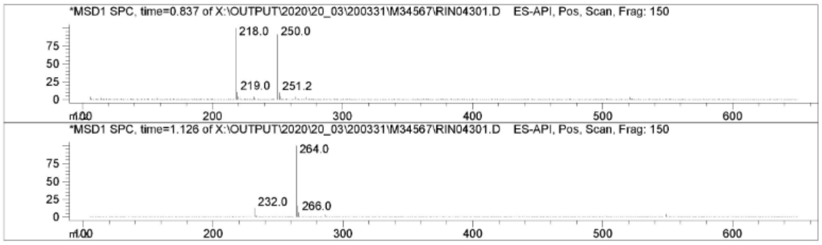

#	Signal	R.Time	Area %
1	ADC1 A, ELSD	1.201	100.000

#	Signal	R.Time	Area %
1	DAD1 A, Sig=215,8 Ref=off	0.751	0.408
2		0.808	2.539
3		0.986	0.027
4		1.011	0.652
5		1.050	0.396
6		1.084	95.978

#	Signal	R.Time	Area %
1	DAD1 B, Sig=241,8 Ref=off	0.751	0.339
2		0.808	3.169
3		1.011	0.467
4		1.050	0.812
5		1.085	95.215

Figure S27. LC/MS data for the reaction product of case 9 (Table 1).

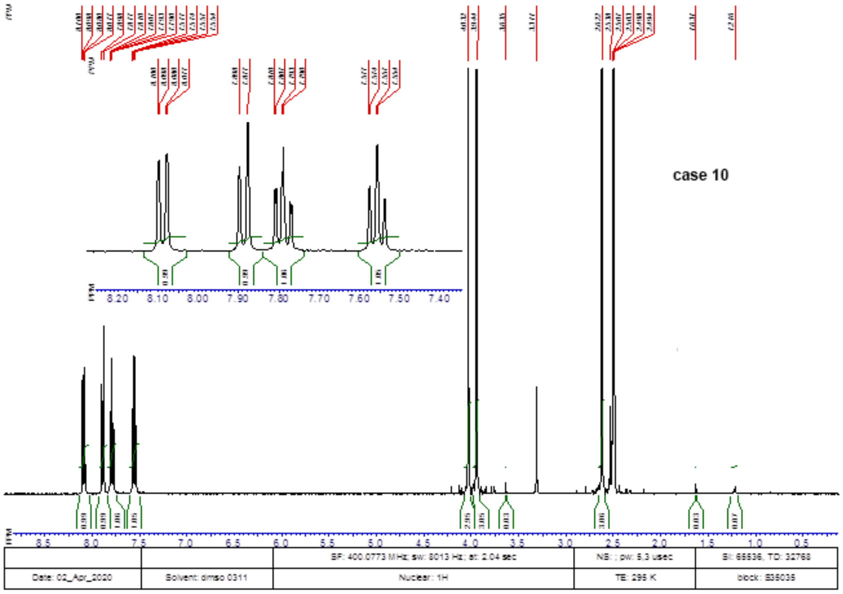

Figure S28. ^{1}H NMR spectrum (400 MHz, DMSO-d6) of the reaction product of case 10 (Table 1).

-.o.-Syntez Purity Report -.o.-

```
Agilent 1100 LC/MSD                              Mobile Phase:A-H2O+0.1HCOOH;B-MeCN+0.1HCOOH
Diodearrey G1315B (DAD1A-215nm; DAD1B-240nm)     Separation column: 97%
Mass Quad  G1956B  (MSD1-Pos, MSD2-Neg)          Rapid Resolutionn HT Cartige 4.6x30mm,
ELSD Altech 3300  (ADC1 A, ELSD)                 1.8-Micron, Zorbax SB-C18
```

case 10

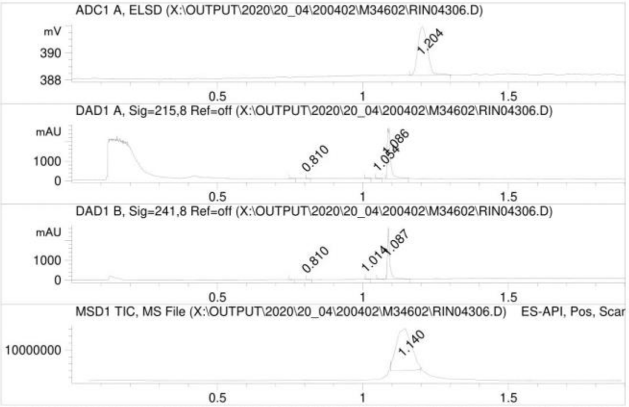

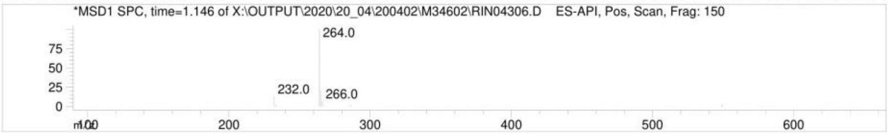

#	Signal	R.Time	Area %
1	ADC1 A, ELSD	1.204	100.000

#	Signal	R.Time	Area %
1	DAD1 A, Sig=215,8 Ref=off	0.753	0.541
2		0.810	0.250
3		1.014	0.572
4		1.054	0.660
5		1.086	97.976

#	Signal	R.Time	Area %
1	DAD1 B, Sig=241,8 Ref=off	0.753	0.595
2		0.810	0.150
3		1.014	0.545
4		1.052	1.323
5		1.087	97.386

#	Signal	R.Time	Area %
1	MSD1 TIC, MS File	1.140	100.000

Figure S29. LC/MS data for the reaction product of case 10 (Table 1).

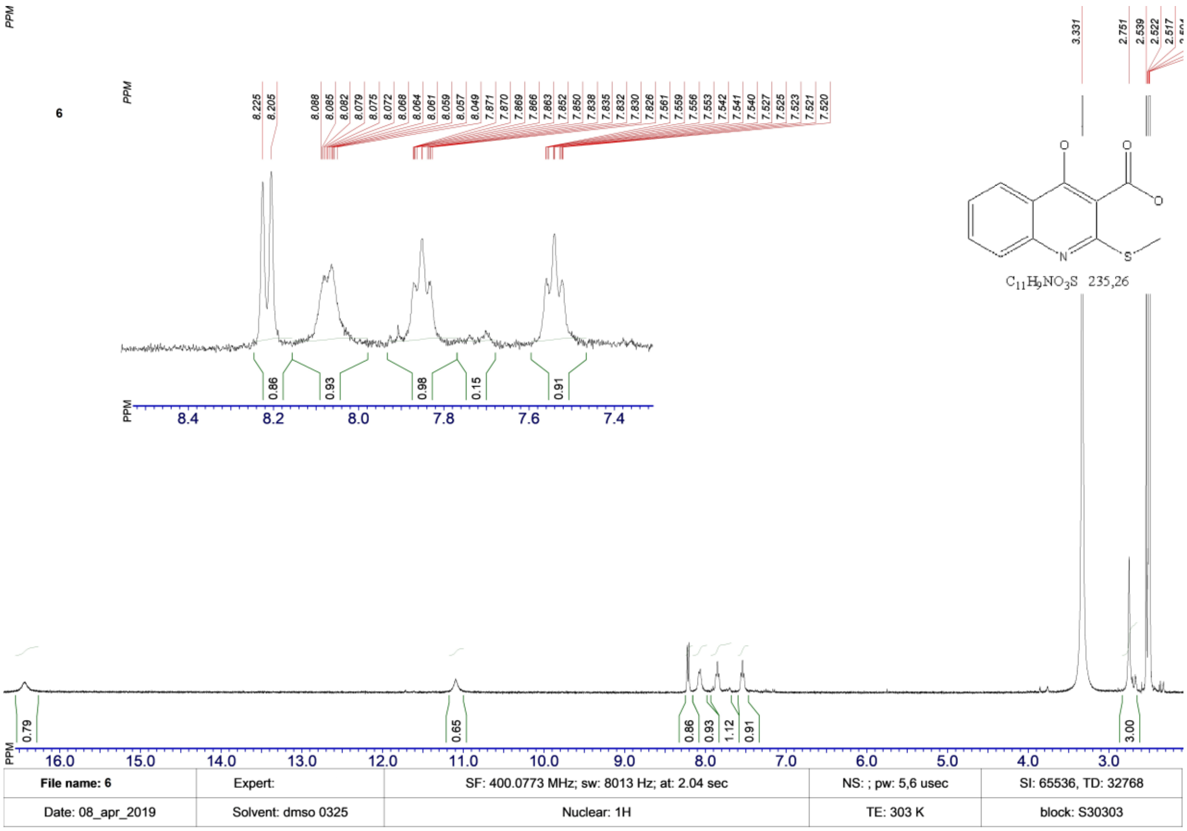

Figure S30. ^{1}H NMR spectrum (400 MHz, DMSO-d6) of 4-hydroxy-2-(methylthio)quinoline-3-carboxylic acid **6**.

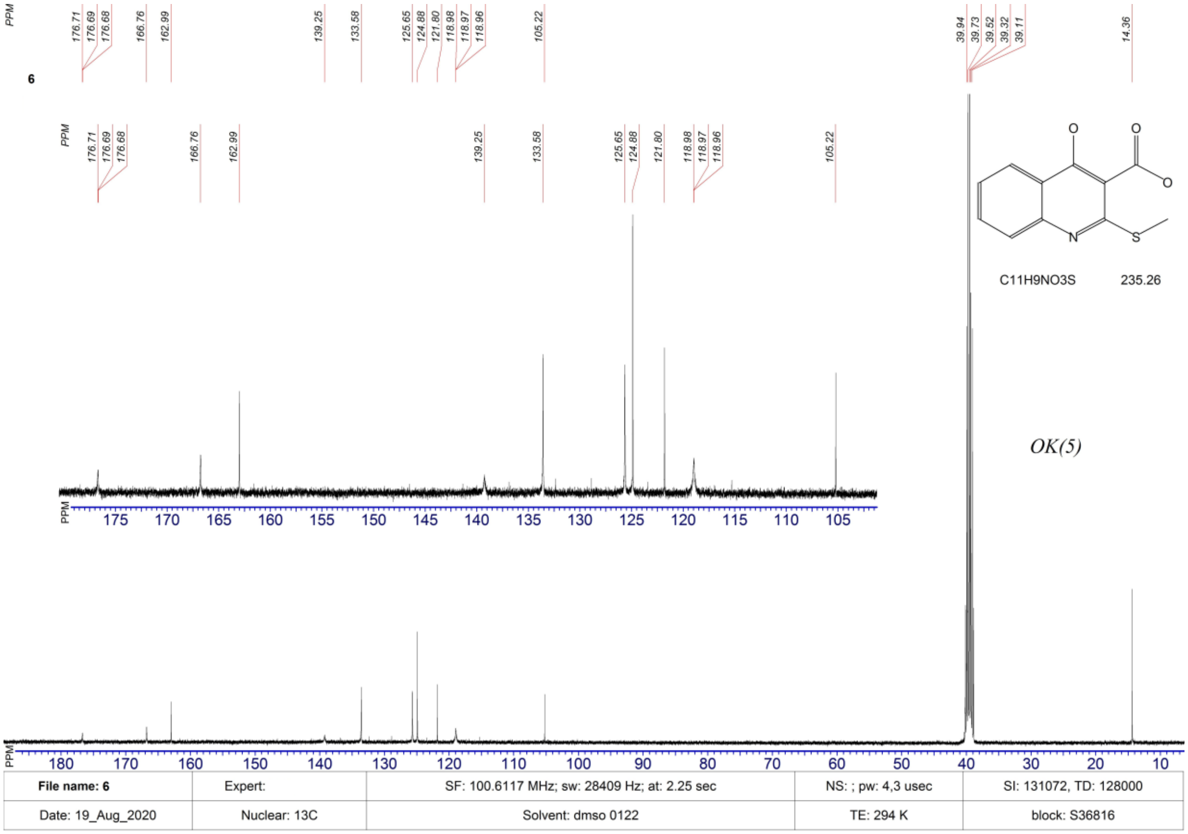

Figure S31. ^{13}C NMR spectrum (100 MHz, DMSO-d6) of 4-hydroxy-2-(methylthio)quinoline-3-carboxylic acid **6**.

```
                        -.o.-Syntez Purity Report -.o.-
Agilent 1100 LC/MSD                                    Mobile Phase:A-H2O+0.1HCOOH;B-MeCN+0.1HCOOH
Diodearrey G1315B (DAD1A-215nm; DAD1B-241nm)           Separation column:
Mass Quad  G1956B (MSD1-Pos,    MSD2-Neg)              Rapid Resolutionn HT Cartige 4.6x30mm,
ELSD Altech 3300  (ADC1 A, ELSD)                       1.8-Micron, Zorbax SB-C18
----------------------------------------------------------------------------------------------->
```

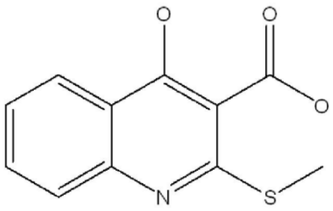

Molecular Weight: 235,26

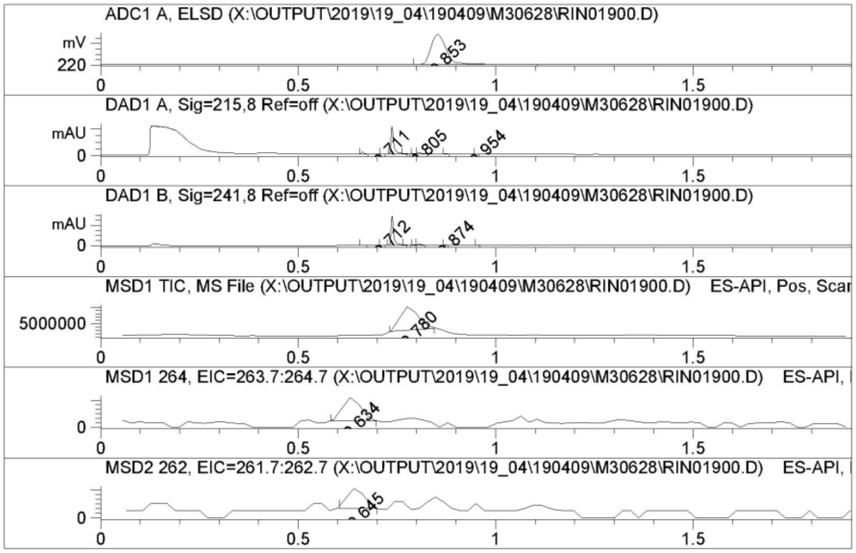

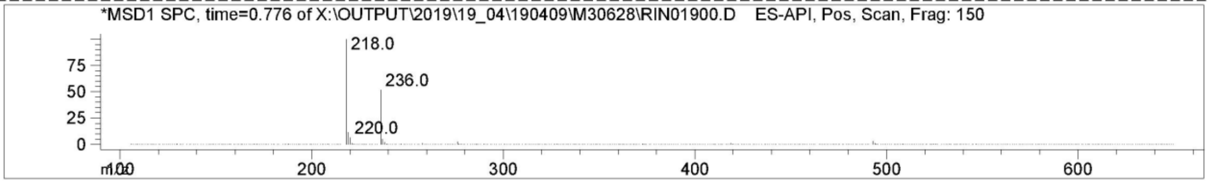

#	Signal	R.Time	Area %
1	ADC1 A, ELSD	0.853	100.000

#	Signal	R.Time	Area %
1	DAD1 A, Sig=215,8 Ref=off	0.663	6.440
2		0.711	0.628
3		0.738	84.932
4		0.769	0.457
5		0.792	0.747
6		0.805	5.532
7		0.874	1.005
8		0.954	0.261

#	Signal	R.Time	Area %
1	DAD1 B, Sig=241,8 Ref=off	0.663	1.292
2		0.712	0.613
3		0.738	92.563
4		0.768	0.417
5		0.793	0.826
6		0.805	3.586
7		0.874	0.215
8		0.954	0.488

#	Signal	R.Time	Area %
1	MSD1 TIC, MS File	0.780	100.000

Figure S32. LC/MS data for 4-hydroxy-2-(methylthio)quinoline-3-carboxylic acid **6**.
